# Global transcriptome analysis of *Huperzia serrata* and identification of critical genes involved in the biosynthesis of huperzine A

**DOI:** 10.1186/s12864-017-3615-8

**Published:** 2017-03-22

**Authors:** Mengquan Yang, Wenjing You, Shiwen Wu, Zhen Fan, Baofu Xu, Mulan Zhu, Xuan Li, Youli Xiao

**Affiliations:** 1 CAS Key Laboratory of Synthetic Biology, CAS Center for Excellence in Molecular Plant Sciences, Institute of Plant Physiology and Ecology, Shanghai Institutes for Biological Sciences, Chinese Academy of Sciences, Shanghai, 200032 China; 2 CAS-JIC Centre of Excellence in Plant and Microbial Sciences, Shanghai, 200032, China; 3 University of Chinese Academy of Sciences, Beijing, 100039 China

**Keywords:** Transcriptome, *Huperzia serrata*, Biosynthetic pathway, Huperzine A, Lycopodium alkaloid

## Abstract

**Background:**

*Huperzia serrata* (*H. serrata*) is an economically important traditional Chinese herb with the notably medicinal value. As a representative member of the Lycopodiaceae family, the *H. serrata* produces various types of effectively bioactive lycopodium alkaloids, especially the huperzine A (HupA) which is a promising drug for Alzheimer’s disease. Despite their medicinal importance, the public genomic and transcriptomic resources are very limited and the biosynthesis of HupA is largely unknown. Previous studies on comparison of 454-ESTs from *H. serrata* and *Phlegmariurus carinatus* predicted putative genes involved in lycopodium alkaloid biosynthesis, such as lysine decarboxylase like (LDC-like) protein and some CYP450s. However, these gene annotations were not carried out with further biochemical characterizations. To understand the biosynthesis of HupA and its regulation in *H. serrata*, a global transcriptome analysis on *H. Serrata* tissues was performed.

**Results:**

In this study, we used the Illumina Highseq4000 platform to generate a substantial RNA sequencing dataset of *H. serrata*. A total of 40.1 Gb clean data was generated from four different tissues: root, stem, leaf, and sporangia and assembled into 181,141 unigenes. The total length, average length, N50 and GC content of unigenes were 219,520,611 bp, 1,211 bp, 2,488 bp and 42.51%, respectively. Among them, 105,516 unigenes (58.25%) were annotated by seven public databases (NR, NT, Swiss-Prot, KEGG, COG, Interpro, GO), and 54 GO terms and 3,391 transcription factors (TFs) were functionally classified, respectively. KEGG pathway analysis revealed that 72,230 unigenes were classified into 21 functional pathways. Three types of candidate enzymes, LDC, CAO and PKS, responsible for the biosynthesis of precursors of HupA were all identified in the transcripts. Four hundred and fifty-seven CYP450 genes in *H. serrata* were also analyzed and compared with tissue-specific gene expression. Moreover, two key classes of CYP450 genes BBE and SLS, with 23 members in total, for modification of the lycopodium alkaloid scaffold in the late two stages of biosynthesis of HupA were further evaluated.

**Conclusion:**

This study is the first report of global transcriptome analysis on all tissues of *H. serrata*, and critical genes involved in the biosynthesis of precursors and scaffold modifications of HupA were discovered and predicted. The transcriptome data from this work not only could provide an important resource for further investigating on metabolic pathways in *H. serrata*, but also shed light on synthetic biology study of HupA.

**Electronic supplementary material:**

The online version of this article (doi:10.1186/s12864-017-3615-8) contains supplementary material, which is available to authorized users.

## Backgroud

*Huperzia serrata* (*H. serrate*) is a model member of the *Huperzia* (*Phlegmariurus*) genus which belongs to the plant Family Lycopodiaceae, with a total of about 500 species worldwide [1, 2]. The whole plant of *H. serrata,* named *Qian Ceng Ta* (in Chinese), is one of the oldest medicinally important traditional Chinese herbs since 739 (during the Tang Dynasty) and has been extensively used for the treatment of a number of ailments, including contusions, strains, swellings, schizophrenia, myasthenia gavis and organophosphate poisoning [3]. These pharmaceutical applications of *H. serrata* are mainly due to its biologically active lycopodium alkaloids. The four classes of lycopodium alkaloids with diverse chemical structures include lycopodine-type, lycodine-type, fawcettimine-type, and a set of miscellaneous-type compounds have been isolated from the *H. serrata,* which makes it a unique model plant for studying the biosynthesis of lycopodium alkaloids [4].

**Fig. 1:**
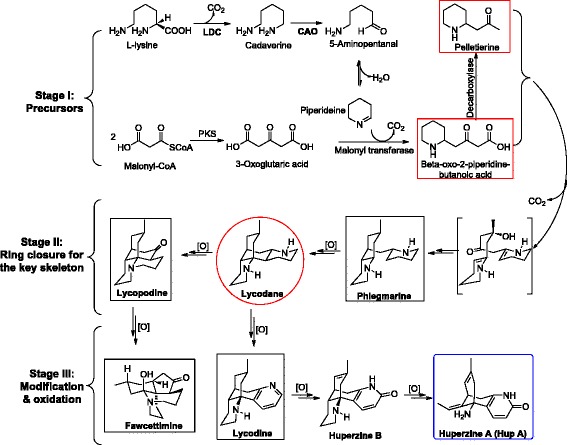
The proposed biosynthetic pathway of HupA and classification of lycopodium alkaloids in *H. serrata*

Among the lycopodium alkaloids, Huperzine A (HupA), one of the pyridine skeleton lycodine, was first isolated from *H. serrata* in 1986 [5] and was found to possess potent acetylcholine esterase inhibition (AChEI) [6, 7]. Since then, HupA had been clinically used for the treatment of Alzheimer’s disease (AD) for years in China. Due to the medicinal importance of *H. serrata* and its economic value of bioactive ingredients, like HupA, this plant has been extensively harvested and is in danger of extinction in China. To overcome the issue, a lot of efforts have been put on plant cultivation and tissue culture for *H. serrata* production, but these approaches are rarely successful. Even the first transcriptomic data generated from 454-GS platform have been released for 6 years [8, 9], there are less than 90 nucleotide sequences are available in the National Center for Biotechnology Information (NCBI) databases (December, 2016). Using homologous cloning strategy, there were only three types of enzymes, lysine decarboxylase (LDC) [10, 11], copper amine oxidase (CAO) [12], and type III polyketide synthase (PKS) [13–15] were identified from *H. serrata.* Feeding experiments provided evidence that lycopodium alkaloids were secondary metabolites of lysine [16]. The first two biosynthetic steps for lycopodium alkaloids were generally believed involving the decarboxylation of lysine yields cadaverine catalyzed by LDC followed by the oxidative deamination of the latter to 5-aminopentanal catalyzed by CAO and then spontaneously to piperidine (Fig. 1) [17]. In the secondary stage of biosynthetic pathway, piperideine was reacted with 3-oxoglutaric acid which was generated from malonyl-CoA and followed by decarboxylation then formed pelletierine. Intramolecular tandem cyclization through phlegmarine would produce the key metabolite, lycodane, which contained the unique tetracyclic skeleton. From this basic intermediate, four classes of lycopodium alkaloids would be derived by further modification and oxidation (Fig. 1). This stage of ring cyclization, rearrangement, modification and oxidation were most likely catalyzed by cytochrome P450s (CYP450s).

The limited transcriptomic data hamper the biosynthetic study of active lycopodium alkaloids in *H. serrata* and the biosynthetic pathway of HupA remains to be elucidated. The HiSeq4000 platform is the most efficient for high-throughput next-generation RNA-Seq which was used for transcriptomic profiling of non-model organisms with no available genomic data. In the current study, a global transcriptome analysis was designed to investigate the full gene contents of *H. serrata* and characterize their expression profiles in differentiated tissues (root, stem, leaf, and sporangia). Our work produced a total of 300 million sequence reads (40.1 Gb of clean data), resulting in 181,141 assembled unigenes. Genes involved in the biosynthesis of the HupA precursor and late stage were identified and predicted, the results of current work will serve as a valuable public resource facilitating the synthetic biology research on bioactive lycopodium alkaloids of traditional herbs.

## Results and discussion

### Sample preparation, and Illumina sequencing

**Fig. 2:**
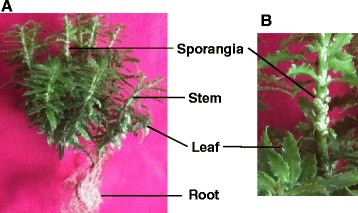
*H. serrata* used in this study. **a** Image of whole-plant. **b** Enlarged image for tissues of sporangia (SP) and leaf (LF). The name of each tissue was labeled with bold words

To characterize the expressed sequences and discover the full gene contents in *H. serrata*, the study was designed to perform deep sequencing on RNA of all four differentiated tissues from it. Root, stem, leaf, and sporangia samples were firstly collected from the first *H. serrata* plant (Hs-1) followed by another two stem and leaf samples from the second one (Hs-2) as complements (Fig. 2). RNA-seq libraries were prepared from the collected tissues, and performed on Illumina HiSeq4000 system with a pair-end read length of 150 base pairs (bp). A total of 300 million raw reads (approximate 45.1 Gb) were generated for all four *H. serrata* tissues, whereas those from roots, stems (2 samples), leaves (2 samples), and sporangias were 7.59 Gb, 7.59 Gb (7.35 Gb from Hs-2), 7.59 Gb (7.35 Gb from Hs-2), and 7.59 Gb, respectively (Table 1).

**Table 1:** Overview of the sequencing and assembly of transcriptome of *H. serrata*

Items	No. of reads (bytes)	No. of bases (bp)
Leaf-1 (from Hs-1)	50,627,568	7,594,135,200
Stem-1 (from Hs-1)	50,627,508	7,594,126,200
Root-1 (from Hs-1)	50,627,472	7,594,120,800
Sporangia-1 (from Hs-1)	50,627,354	7,594,103,100
Leaf-2 (from Hs-2)	48,994,336	7,349,150,400
Stem-2 (from Hs-2)	48,994,084	7,349,112,600
Total raw data	300,498,322	45,074,748,300
Total high-quality data	267,314,012	40,097,101,800
Average length of unigenes	1,211 bp	
Unigenes ≥ 300 bp	181,141	219,520,611
N50 (bp)	2,488	

### *De novo* transcriptome assembly, gene expression comparison among tissues, and comparison with previous 454-ESTs report

**Fig. 3:**
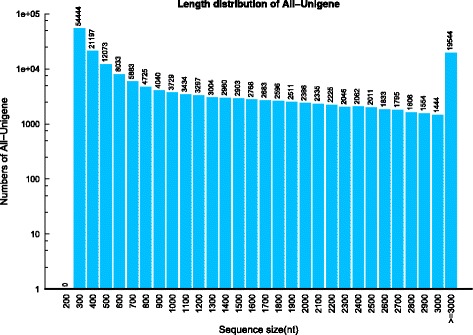
Length distribution of assembled unigenes. Out of the 181,141 unigenes, 105,516 unigenes (58.25%) were longer than 500 bp, and 70746 unigenes (39.06%) were longer than 1.0 kb

After reads filtering to remove adaptor sequences and low-quality reads, high-quality clean reads from the four tissues were pooled together for *de novo* assembly using Trinity [18], and a total of 830,623 contigs were generated with a mean size of 812 bp. Hence, 181,141 unigenes were obtained with an average size of 1,211 bp ranging from 301 bp to 3,000 bp in size. In total, 105,516 unigenes (58.25%) were longer than 500 bp, and 70,746 unigenes (39.06%) were longer than 1.0 kb (Fig. 3).

**Fig. 4:**
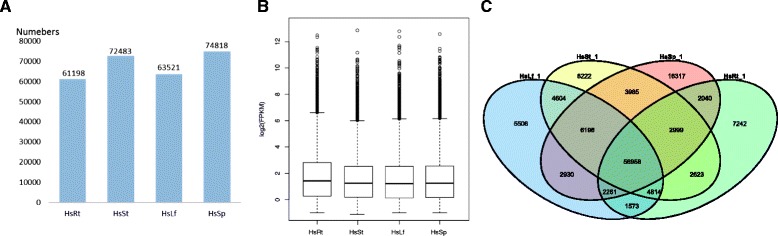
Unigenes expressed number and level on different tissues. **a** The number of expressed genes (FPKM ≥ 0.5) in each tissue was labeled over the bars. **b** Expressional levels of unigenes of all the tissues. **c** Venn diagram of expressed unigenes in four tissues. A total of 130,297 unigenes (71.9%) were expressed, of which 56,958 genes were common expressed in all four tissues. Unigenes with FPKM ≥ 0.5 were considered as expressed in each tissue

In order to get the gene expression profiles of each individual tissue, all the data from different tissues was firstly analyzed by cluster dendrogram (Additional file 1). Based on the relationship between the six data sets, four tissue data sets from the same plant (Hs-1) were picked for following tissue-specific analysis and further mapped back to the assembled unigenes using bowtie2 with their clean reads [19]. While the sporangia tissue has the highest numbers of gene expressed, all the four tissues shared a similar distribution in gene expression level (Fig. 4). There were 130,297 unigenes (71.9%) expressed above the threshold level (fragment per kilobase per million mapped reads (FPKM) ≥ 0.5) in the four tissues, of which 56,958 genes were common expressed in all the four tissues (Fig. 4c). Generally, the sporangia expressed the largest number of tissue-specific unigenes (18,317), followed by the stem (8,222), root (7,242), and leaf (5,506).

A total of 267,314,012 high quality reads is three orders of magnitude greater than the previous reported from *H. serrata,* in which 140,930 high quality reads generated from 57 Mb data using Roche 454 GS FLX Titanium system (Additional file 2) [8, 9]. The reads generated from 454 platform were assembled to 14,085 contigs using GS De Novo Assembler software v2.0.01 (454 Life Sciences, Roche) with an average length of 608 bp. With the higher throughput of Illumina Hiseq platform, more and longer contigs were produced. Our clean reads were assembled to 830,623 contigs, with the average length is 812 bp. 181,141 unigenes were generated and 58.25% of them were annotated at last, to some extent, showing the disadvantage of short-read sequence assembly was avoided by more sequence data.

### Function annotation of transcriptome for *H. serrata*

**Fig. 5:**
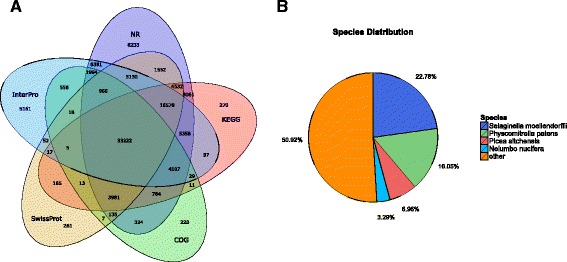
Results summary for the sequence homology search against the NCBI NR database. **a** Venn diagram of transcripts detected in NR, COG, KEGG, Swissprot and Interpro. **b** Distribution of annotated species

All assembled unigenes were annotated with 7 functional databases (NCBI non-redundant nucleotide database (Nr), NCBI non-redundant protein database (Nt), Gene Ontology databases (GO), Clusters of Orthologous Groups of proteins database (COG), Kyoto Encyclopedia of Genes and Genomes (KEGG), Swiss Institute of Bioinformatics (Swiss-Prot), and InterPro) (Table 2). In total, 58.25% (105,516) annotated sequences were identified, which was higher than the 454-EST level reported (44.3%) [8, 9]. These assembled transcripts were mapped to the 7 public databases, and the amount of transcripts which were aligned to each database were shown in Fig. 5a. Among the annotated sequences, the species with the highest number of best hits were *Selaginella moellendorffii* (22.78% matched genes) and *Physcomitrella patens* (16.05% matched genes) (Fig. 5b). These results are consistent since *S. moellendorffii* and *P. patens* are the species closest to *H.serrata* with sequenced genomes, in which both of them are members of ancient vascular plant lineage, and *P. patens* is a lycophyte, the oldest extant division of the vascular plants.

**Table 2:** Summary of unigenes annotations

Database	Total unigenes	Annotated unigenes	Percentage (%)
Nr	181,141	94,338	52.08
Nt	181,141	52,626	29.05
Swiss-Prot	181,141	64,634	35.68
KEGG	181,141	72,230	39.88
COG	181,141	46,372	25.60
Interpro	181,141	77,671	42.88
GO	181,141	45,780	25.27
Overall	181,141	105,516	58.25

#### GO and KEGG classification

**Fig. 6:**
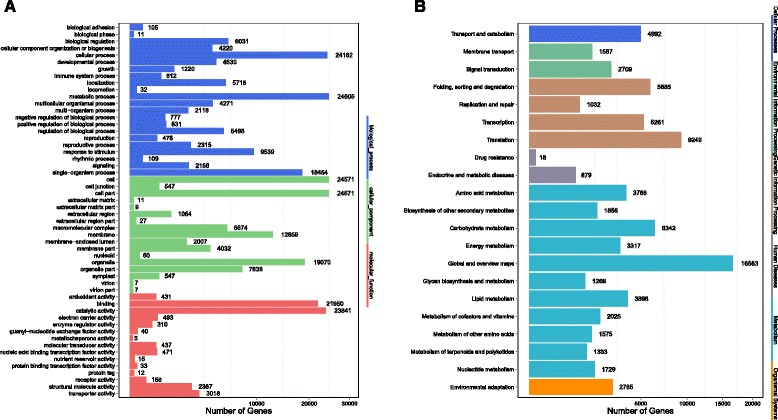
GO and KEGG analysis. **a** Histogram of GO classifications. The results are summarized in the following three main categories: (1) biological process, (2) cellular component and (3) molecular function. **b** Pathway assignment based on KEGG. The bottom x-axis indicates the number of genes in a specific category. The left y-axis indicates the clustered function groups, the right y-axis indicates the specific category of genes in the main category

The function annotation was conducted on the basis of homology. GO assignments were used to predict the functions of *H.serrata* unigenes by classifying them into three major categories: biological process, cellular component and molecular function (Fig. 6a). Based on sequence homology, the 45,780 unigenes annotated in the GO database were categorized into 54 functional groups. Among these groups, “cellular process and metabolic process”, “cell and cell part”, and “binding and catalytic activity” terms were dominant within each of these categories, respectively. Additionally, we noticed that many genes were classified into the “single-organism process”, “organelle” and “membrane” groups, whereas only a few genes were classified into the “extracellular matrix part”, “virion” and “metallochaperone activity” groups. The high percentage of genes from the “metabolic process” (24,605) and “catalytic activity” (23,841) might serve as good candidates for identification of novel genes that participated in the lycopodium biosynthesis pathway. KEGG is a classification method based on pathways for systematic analysis of gene function in terms of the networks of gene products. To identify biochemical pathways, 72,230 genes were mapped to 136 KEGG pathways which were classified into six main categories (Fig. 6b). Most genes were annotated with metabolic function (60.47%, 43,674), followed by “genetic information processing” (29.66%, 21,427). The pathways with most representation were “global and overview maps” (16,563 unigenes, 22.93%), “translation” (9,249 unigenes, 12.80%), and “carbohydarte metabolism” (6,342 unigenes, 8.78%).

These annotation and classifications provided a resource for investigating specific pathways in *H. serrata*, such as lycopodium alkaloids biosynthetic pathway. Lycopodium alkaloids are lysine derivated alkaloids, therefore, the 3,768, 1,858, and 1,333 unigenes clustered into “amino acid metabolism”, “biosynthesis of other secondary metabolites”, and “metabolism of terpenoids and polyketides” might potentially be involved in the biosynthesis and metabolism of lycopodium alkaloids.

#### Transcription factor (TF)

**Fig. 7:**
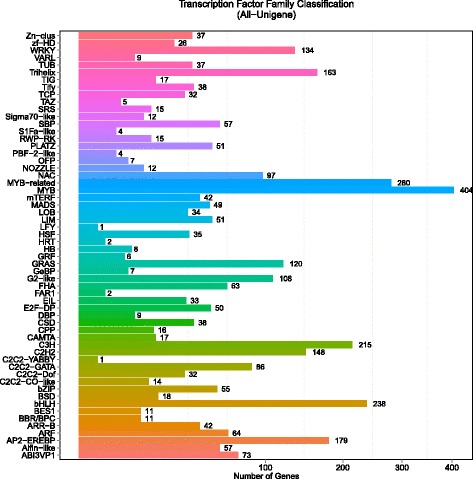
Transcription Factor (TF) family classification of unigenes. Number of unigenes related to TFs in each families, MYB, MYB-related, bHLH, and C3H proteins were the most abunant

With functional annotation results, we detected 94,510 CDS (coding DNA sequence) firstly, and continually found 18,139 CDS predicted by ESTScan with the remaining unigenes. We also detected 1,478 SSR which distributed on 26,579 unigenes, and predicted 2,975 TF coding unigenes with family classification (Fig. 7 and Additional file 3). Among the TF types, MYB (404 members), MYB-related (280 members), bHLH (238 members) and C3H (215 members) proteins were the most abundant above 200 members, with the largest (MYB) having 404 members in comparison with 155 and 197 MYB genes in rice and *Arabidopsis*, respectively [20]. Functions of MYB proteins are involved in secondary metabolism, regulation of gene expression and response to environmental stresses [21].

### Genes involved in the biosynthesis of lycopodium alkaloids and HupA

To identify active biological pathways in *H. serrata*, the sequences were mapped to the reference pathways in the KEGG. Most metabolic related genes were involved in primary metabolism, such as carbohydrate metabolism, lipid metabolism, amino acid metabolism, and energy metabolism. The 1,858 genes were grouped in the “Biosynthesis of other secondary metabolites” category, which were important contributors to highly valuable secondary metabolite biosynthesis. Especially 140 genes were mapped to the “tropane, piperidine and pyridine alkaloid biosynthesis”. The 119 genes were mapped to the “isoquinoline alkaloid biosynthesis” would be useful for defining metabolic pathways and metabolic genes for lycopodium alkaloids synthesis in *H. serrata* (Fig. 6b)*.*

**Fig. 8:**
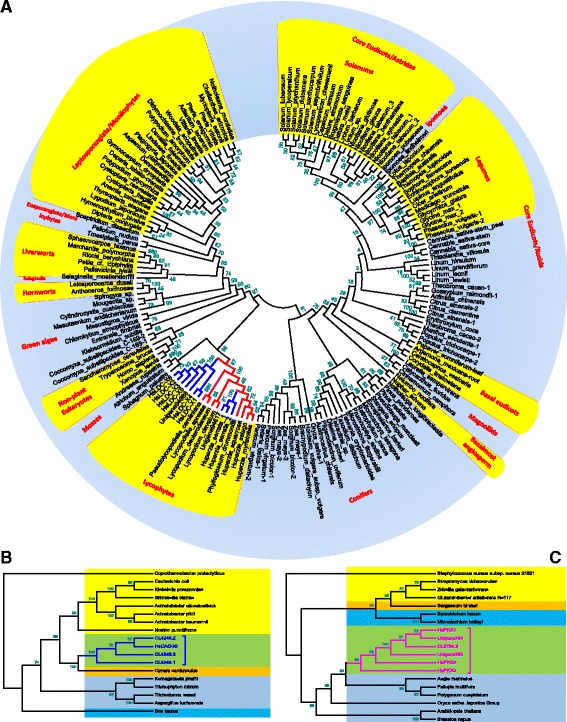
Phylogenetic analysis of LDC, CAO, PKS. Unrooted neighbor-joining phylogenetic tree of selected LDC, CAO, PKS proteins. Bootstrap values (1000 replicates) were shown above each branch. **a** Phylogenetic tree of LDC amino acid sequences from eukaryotes. Phylogenetic tree of CAO (**b**) and PKS (**c**) amino acid sequences. The blue branch lines represented genes in this study and biochemically confirmed [11–15]

As shown in the Fig. 1, the biosynthesis pathway of lycopodium alkaloids as well as HupA, was proposed within three stages, formation of the precursors, ring closure for the tetracyclic basic skeleton, and modification and oxidation. In the first stage of producing necessary precursors starting from lysine and malonyl-CoA as the initiated substrates, it was believed that LDC, CAO, and PKS were involved in the bioconversion. Using degenerate primers strategy based on the sequence homology, these three types of genes/enzymes from *H. serrata* have been identified by us and other research groups followed by biochemically characterization [10–15]. All these genes were also found in our transcriptomic data (Table 3 and Additional file 4 with the detailed sequences). These gene’s homologues and their multiple-tissues specific expression patterns were also analyzed by phylogenetic tree and heat map (Additional file 5). Besides, we performed phylogenetic analysis for LDC, CAO, PKS with their reported homologues and accession numbers of selected genes (Fig. 8a-c and Additional file 6). It’s clear that the eukaryotic LDCs were monophyletic origin from the phylogenetic analysis which was consistent to previous study (Fig. 8a) [10]. As Lycophyte, Angiosperm, and Legume were responsible for biosynthesis of lycopodium, nuphar, and quinolizidine alkaloids, respectively, the LDC clade formed from these three distinct plant lineages were distant from each other which indicated a convergent evolution of the Lys-derived alkaloid production. In conclusion, these genes were located in the lower creatures. Generally, these three types of biochemically confirmed genes were either exact same or had high identity to the corresponding transcripts from the phylogenetic tree analysis. The newly discovered homologue genes provide more enzymatic parts in the toolbox for synthetic biology usage in the biosynthesis of precursors of HupA.

**Table 3:** Possible unigenes and encoding enzymes involved in biosynthesis of HupA precursor

Gene	Degenerate primes method	Unigenes from transcriptomic data
LDC	AB915696.1, AB915697.1^[10]^ and HsLDC-X1 to -X6^[11]^	unigene96617 and unigene94988
CAO	HsCAO ^[12]^	CL4248.1, CL4248.2, CL4248.3
PKS	HsPKS1 (ABI94386.1)^[13]^, HsPKS2^[14]^, and HsPKS3^[15]^	unigene393, CL2724.2, and unigene394

**Fig. 9:**
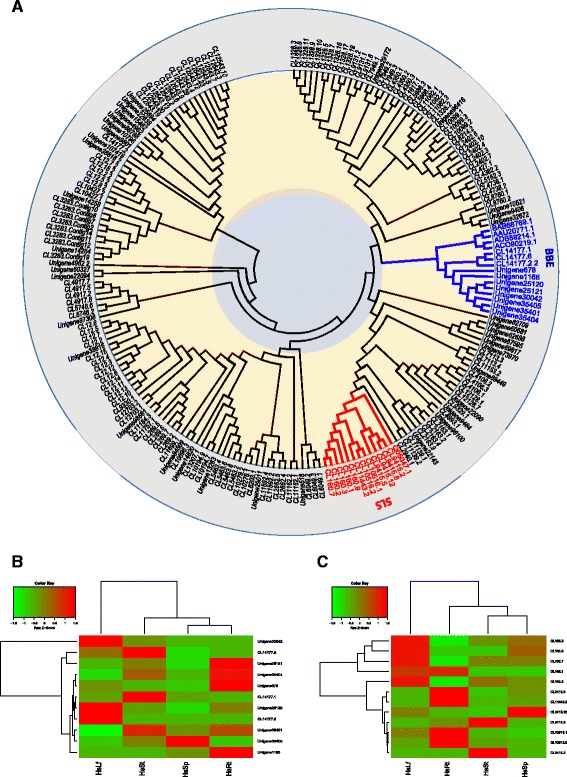
Phylogenetic analysis of CYP450s and the heat map of BBE and SLS. **a** Phylogenetic analysis of 199 full-length CYP450s. The tissue-specific heat map of BBE (**b**) and SLS (**c**). The detail information about all the CYP450s including BBE and SLS were listed in Additional file 7–9

In the late two stages of biosynthesis of lycopodium alkaloids and HupA, most of steps were proposed through oxidative modifications which indicated that CYP450s were candidates. To identify novel genes encoding enzymes possibly associated with the catalytic modification, 457 putative CYP450 sequences with 199 full-length ones were obtained which was about 5 fold greater than the 96 CYP450 members in 454-ESTs data [9]. These P450 sequences were annotated and classified into different CYP families by using phylogenetic analysis (Fig. 9a and Additional file 7). The middle stage of conduction of the key scaffold, like phlegmarine and lycodane, through intramolecular tandem cyclization with new C-C bond formation, shares a similar formation of the methylenedioxy bridge in berberine synthesis with the gene encoded by AAU20771.1 from *Thalictrum tuberosum* [22]. With this gene as the probe, a phylogenetic tree was generated and 11 homologues were identified as the berberine bridge enzyme (BBE) (Additional file 7 and Additional file 8). The heat map was also analyzed with differential tissues (Fig. 9b). In addition, It was also noteworthy that 12 CYP450s belong to a group with CYP450 (Q05047.1) encoding the enzyme secologanin synthase (SLS) based on the phylogenetic analysis (Additional file 7 and Additional file 9), which catatlyzed the oxidative cleavage of loganin into secologanin [23]. From the tissue-specific analysis (Fig. 9c), these candidate CYP450s were expressed more abundantly in leaves than other organs, which was consistent with the organ-specific accumulation pattern of lycopodium alkaloids [24]. These two classes of CYP450s may play key roles in biosynthesis of lycopodium alkaloid as well as HupA and will be considered as lead candidates in the late two stages of ring formation and oxidation.

### Validation of RNA-Seq results by qRT-PCR

qRT-PCR was performed to validate four types of differentially expressed genes identified by RNA-Seq in the four tissues of *H. serrata*. With tubulin (Unigene50132) as the internal control, 7 selected genes involved in HupA biosynthesis, including HsLDC, HsCAO, HsPKS and CYP450s (SLS and BBE classes), were evaluated. The validation results were consistent with the gene expression patterns identified by RNA-Seq (Additional file 10). The expression levels of Unigene25121 were slightly higher based on qRT-PCR than RNA-Seq. These results highlighted the fidelity and reproducibility of the RNA-Seq analysis used in the present study.

## Conclusion

We conducted deep RNA-sequencing analysis on four tissues of *H. serrata* and a total of 300 million reads were generated. 181,141 unigenes were assembled, in which nearly 60% were successfully annotated. The data offered a comprehensive coverage of *H. serrata* transcriptome and paved the way for elucidation of the biosynthesis pathway of lycopodium alkaloids, like HupA. The three types of biochemically confirmed enzymes in the biosynthesis of the precursors, LDC, CAO and PKS, were all identified in this study. Moreover, a large number of CYP450s involved in the secondary metabolic pathway were evaluated. We predicted that the BBE and SLS types of CYP450s were involved the ring closed and cleavage in the biosynthesis of HupA. Further studies are needed to elucidate the CYP450s involved in the ring formation and oxidative modification of the biosynthesis of HupA. The study provides valuable resources for bioengineering and synthetic biology study of the lycopodium alkaloids.

## Methods

### Plant materials and treatments

Two independent *H. serrata* plants (Hs-1 and Hs-2) were collected from Xiangxi, Hunan, China, in December 2015 and identified by Dr. Zhu Mulan. The plants were carefully rinsed in running tap water and soil was removed by hand. Root, stem, leaf, and sporangia, were kept in collection tubes immediately after separated from the plant and immersed in liquid nitrogen, and then stored at -80 °C until further use.

### RNA isolation, cDNA library construction and Illumina sequencing

Total RNA was extracted from four different tissues of *H. serrata,* including root, stem, leaf and sporangia with TIANGEN RNAprep Pure Plant Kit. DNase I was used to digest contaminated DNA. The purified total RNA was quantified using Nanodrop, Agilent 2100, and agrose gel electrophoresis. Oligo(dT) was used to isolate mRNA followed by fragmentation. cDNA was synthesized using the mRNA fragments as templates. Short fragments were purified and resolved with EB buffer for end reparation and single nucleotide A (adenine) addition followed by connected with adapters. The suitable fragments were selected for the PCR amplification. Agilent 2100 Bioanaylzer and ABI StepOnePlus Real-Time PCR System were used in quantification and qualification of the sample library. The cDNA library was sequenced from both of 5’ and 3’ ends on the Illumina HiSeq4000 platform with paired-end sequecing length of 150 bp according to the manufacturer’s instructions.

### De novo assembly and mapping of sequencing reads and analysis

Trinity was used to perform *de novo* assembly with clean reads that PCR duplication removed (in order to improve the efficiency), and Tgicl was used to cluster transcripts to unigenes. After assembly, clean reads were mapped to unigenes using Bowtie2 [19], and then gene expression level was calculated with RSEM [25]. To assess the gene expression abundance, the differentially expressional levels of unigenes in the four tissues were measured by FPKM values, with FPKM ≥ 0.5 used as a cut-off.

### Functional annotation and classification

All assembled unigenes were searched against the Nr database and the SWISS-PROT database using BLASTX. Unigenes were also compared with the COG and KEGG using BLASTX. InterPro domains were annotated by InterProScan5 [23] and functional assignments were mapped onto GO database.

### Phylogenetic analysis

Amino acid sequences were aligned using the CLUSTAL W program and evolution distances were computed using the Poisson correction method, and a Neighor-Joining (NJ) tree was constructed with MEGA6. Bootstrap values which have been converted into the percentage obtained after 1000 replications are given on the branches

### Quantitative real-time PCR

Quantitative real-time PCR (qRT-PCR) amplification was performed to validate the RNA-seq data with the designed primers (Additional file 10). The experiment was conducted with CFX Real-Time PCR Detection System (BIO-RAD, USA) using SYBR® Premix Ex Taq™ kit (TaKaRa), and it was repeated three times. The mean value of three replicates was normalized using Tubulin (unigen50132) as the internal control. PCR mixtures (final volume, 25.0 μL) contained 200 ng of cDNA, 0.200 μM each primer, 8.00 μL of sterile water, and 12.5 μL of SYBR Green Premix Ex Taq (TakaRa). The conditions for amplification were described as follows: 10 min denaturation at 95 °C, 40 cycles of 95 °C for 10 s, 57 °C for 20 s, and 72 °C for 20 s. Melting curves were determined ranging from 60 °C to 95 °C at 0.5 °C/min.

## Additional files

Additional file 1: All the multiple-tissues specific data from *H. serrata* analyzed by cluster dendrogram. (PDF 81 kb)

Additional file 2: Summary of *H. serrata* RNA seq by two Next-Generation Sequencing Systems (PDF 44 kb)

Additional file 3: List of transcription factors (TFs) obtained from *H. serrata* transcriptome. (XLS 194 kb)

Additional file 4: The ORF or amino acid sequences of three types (LDC, CAO, PKS) of enzymes identified from *H. serrata* and found in the transcriptome. LDC (HsLDC-X1, HsLDC-*X*2, HsLDC-X3, HsLDC-X4, HsLDC-X5, HsLDC-X6, AB915697.1, AB915696.1, Unigene94617, Unigene94988); CAO (HsCAO, CL4248.1, CL4248.2, CL4248.3); PKS (HsPKS1 (ABI94386.1), HsPKS2, HsPKS3, Unigene393, Unigene394, CL2724.2). (PDF 63 kb)

Additional file 5: LDC’s, CAO’s, and PKS’s multiple-tissues specific expression patterns analysis by heat map. (PDF 63 kb)

Additional file 6: Selected genes for LDC’s, CAO’s, and PKS’s phylogenetic analysis. (PDF 66 kb)

Additional file 7: Unrooted neighbor-joining phylogenetic tree of selected CYP450s (Bootstrap values (1000 replicates)). (PDF 45 kb)

Additional file 8: The amino acid sequences of 11 homologues identified as BBE. (PDF 33 kb)

Additional file 9: The amino acid sequences of 12 homologues identified as SLS. (PDF 35 kb)

Additional file 10: qRT-PCR expression analyses of HsLDC, HsCAO, HsPKS, and CYP450s. (PDF 36 kb)

## Abbreviations

CAO: Copper amine oxidase
CDS: Coding DNA sequence
COG: Clusters of orthologous groups of proteins database
CYP450s: Cytochrome P450s
FPKM: Fragment per kilobase per million mapped reads
GO: Gene ontology databases
KEGG: Kyoto encyclopedia of genes and genomes
LDC: Lysine decarboxylase
NCBI: National center for biotechnology information
Nr: NCBI non-redundant nucleotide database
Nt: NCBI non-redundant protein database
PKS: Polyketide synthase
Swiss-Prot: Swiss institute of bioinformatics
