# Supplementary figures and images for: Global transcriptome analysis of Huperzia serrata and identification of critical genes involved in the biosynthesis of huperzine A
Source: BMC Genomics. 2017 Mar 22;18:245. doi: 10.1186/s12864-017-3615-8 (PMC5361696; doi:10.1186/s12864-017-3615-8)

All the data from different tissues analyzed by cluster dendrogram

Cluster Dendrogram

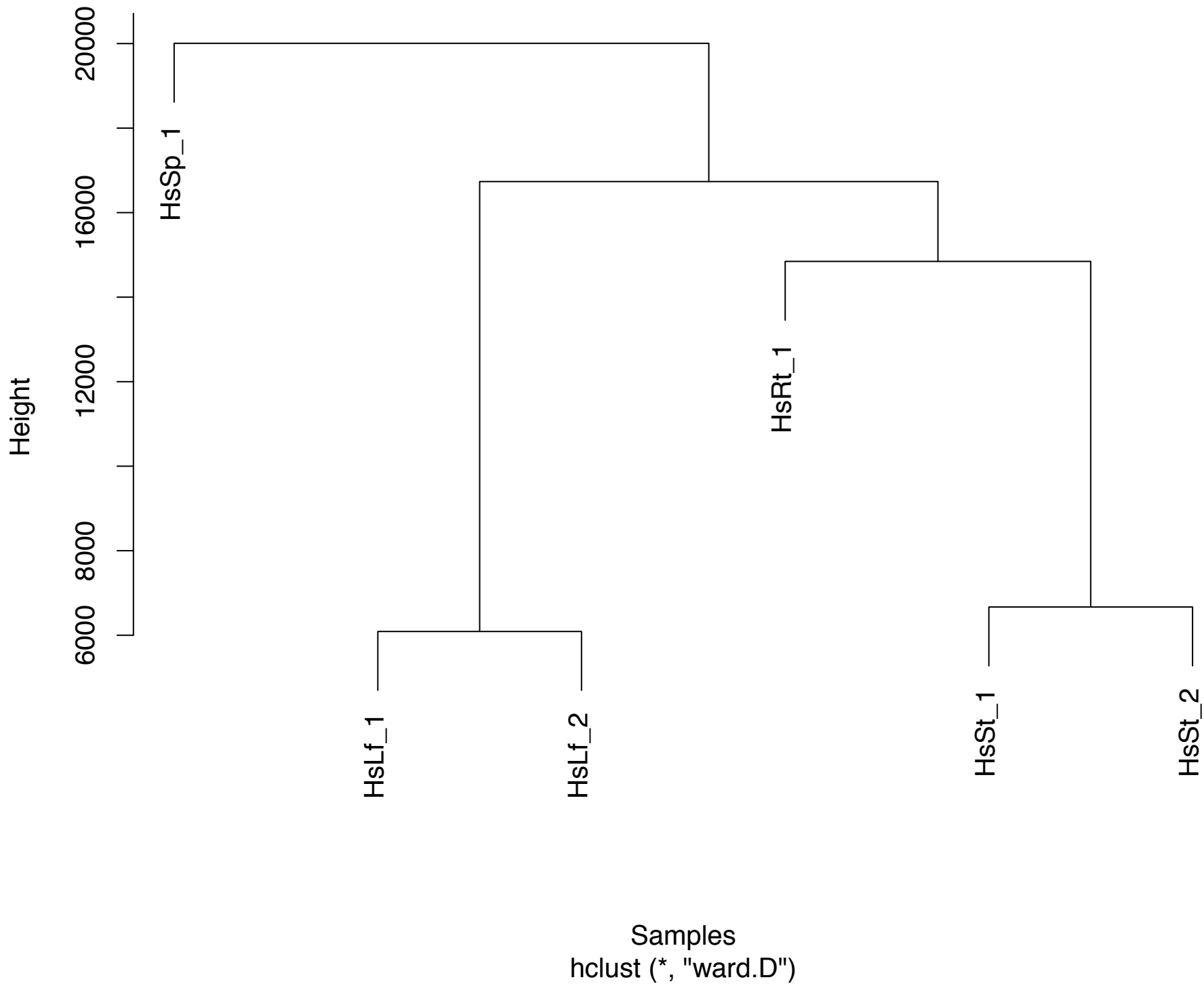

Supplement: Supplementary file 1 — All the multiple-tissues specific data from H. serrata analyzed by cluster dendrogram. (PDF 81 kb) [file 12864_2017_3615_MOESM1_ESM.pdf]

CYP450 phylogenetic analysis

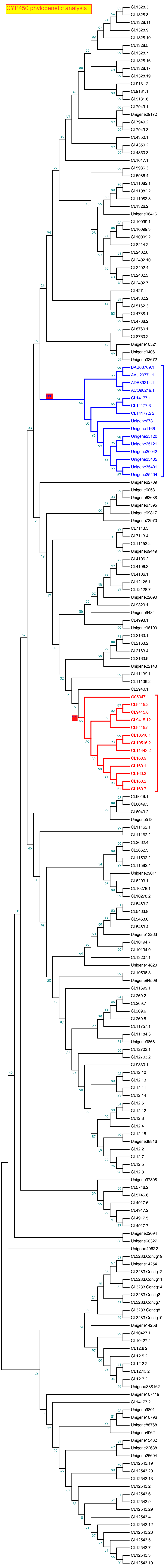

Supplement: Supplementary file 7 — Unrooted neighbor-joining phylogenetic tree of selected CYP450s (Bootstrap values (1000 replicates)). (PDF 45 kb) [file 12864_2017_3615_MOESM7_ESM.pdf]
