# Supplementary material for: Global transcriptome analysis of Huperzia serrata and identification of critical genes involved in the biosynthesis of huperzine A
Source: BMC Genomics. 2017 Mar 22;18:245. doi: 10.1186/s12864-017-3615-8 (PMC5361696; doi:10.1186/s12864-017-3615-8)
Supplement: Supplementary file 2 — Summary of H. serrata RNA seq by two Next-Generation Sequencing Systems (PDF 44 kb) [file 12864_2017_3615_MOESM2_ESM.pdf]

Summary of *H. serrata* RNA seq by two Next-Generation Sequencing Systems

**Sequencing Platform**	**Illumina Hiseq4000**	**Roche 454-GS FLX Titanium**	**Difference (fold)**
Total bases	40.1 Gb	57.0 Mb	720
NO. of HQ reads	267,314,012	140,930	**1897**
Average read length	150 bp	405 bp	0.37
NO. of contigs	830,623	14,085	59.0
Average contig length	812±58 bp	608±460 bp	1.33
No. of contigs larger than 500 bp	359,475 (43.3%)	8,948 (63.5%)	40.2
No. of singletons	115,520	22,678	5.09
Average singleton length	711 bp	351 bp	2.02
No. of singletons above 200 bp	114,715	16,879	6.80
No. of unique putative transcripts	181,141	36,763	**4.93**
Annotated sequences	105,516 (58.2%)	16,274 (44.3%)	**6.48**
