# Supplementary material for: Global transcriptome analysis of Huperzia serrata and identification of critical genes involved in the biosynthesis of huperzine A
Source: BMC Genomics. 2017 Mar 22;18:245. doi: 10.1186/s12864-017-3615-8 (PMC5361696; doi:10.1186/s12864-017-3615-8)
Supplement: Supplementary file 4 — The ORF or amino acid sequences of three types (LDC, CAO, PKS) of enzymes identified from H. serrata and found in the transcriptome. LDC (HsLDC-X1, HsLDC-X2, HsLDC-X3, HsLDC-X4, HsLDC-X5, HsLDC-X6, AB915697.1, AB915696.1, Unigene94617, Unigene94988); CAO (HsCAO, CL4248.1, CL4248.2, CL4248.3); PKS (HsPKS1 (ABI94386.1), HsPKS2, HsPKS3, Unigene393, Unigene394, CL2724.2). (PDF 63 kb) [file 12864_2017_3615_MOESM4_ESM.pdf]

ORF Sequence of LDC, and CAO and amino acid sequences of PKS

1) Lysine decarboxylases (LDC)

>HsLDC-X1

ATGACGTTTGGTCGCATGGAAGCAATTGCTCCTTCCCTGGGCATGGATGAAGTCGTTGCAGCTCGTAGCACT
TTCTTTCAATATTCCACGGATGGAGATGACAACAGCAGCCTGCACAGCATGAATAAAGCTCTAGGTGATTTG
ATTAGAAAGTTAGTGGAGTCCTCAGTCCACCACACTGTCAGCATCATCCCATCTTCATCTCAGAGAGACGTTG
ACATTTTCATCAATAGCACGGTCAATGAAGACAAGCTGCAGGAACCTTTCTTCGTTCTAGACCTCGGCGTGC
TTGTTCGACTGTTCTACGATTGGATCAAGGCTCTTCCAAGGGTGCTGCCTTACTACGCAGTGAAATGCAATCC
GAGTAGACCATTGCTGGCCACCATGGCAGCCCTGGGAGCAGGCTTCGATTGTGCCAGCAAGCTGGAAGTA
GAAGAAGTGCTTGCTCTTGGAGTGGCTCCACAGAGAATCATATTCGCAAATCCATGCAAAATGCCTTCTCAC
ATCAATTATGCTGCATGTAAGGGTGTGCATGTCACCACCTTCGATTCTGAAGGAGAAGTGATGAAGGTGAAG
GCCAATGATCCGAATGCAACTTTACTTCTGCGTCTTAAGACGGACGACGAGAGCTCTAAATGCCCACTGGGG
GTGAAGTATGGAGCACTAATGAGCGAGGTGGATCATCTGCTGCTCGTAGCAACCAATTCTGAGGTTCCTGTG
ATTGGAGTCTCCTTTCATATCGGCAGTGGAGCTTGCGATGCCCAAGCTTACACACACGCCATAGCTGCAGCT
CGATGCGTATTTGATAAAGGAGTGCAGCTAGGCCTGCCACCAATGTCTGTTCTGGACATAGGGGGAGGCTT
CACTGTGGGCACAAATGGAGAGCTGAAGCTGGCGCAAGCTGCAGAAGCAATCAACGCAGCCCTGGATCAG
TACTTCCCACCTGGAATGGGCGTTTCCATCGTTGCAGAGCCAGGACGATTCTTTGCAGAATGCCCTACCACG
CTAGCTACCACCATCTATGGTAAGCGAGTCCGCGGAGAATTAGTAAGAGAATATTGGATCAACGACGGCATC
TATGGCTCTATGAACAATCTCATTCATGACTACGCTGTTCTGAGGCCCCGTCCTTTGGCTTGCGCTTCCCATCA
AACCAATCTCTCCTGCACTGGCCTTATCACCTTTTCCTCTACAGTCTTTGGACCAACCTGCGATTCTTTGGATA
CTGTGCTGAAAGATTATGAGCTACCTGATCTTGCAGACGGAGACTGGATTGTGTTTTCCAACATGGGAGCAT
ACACCCATTGCGCTGGATCCAGCTTCAATGGCTACTCCACTTCTGATATCAGAACATACTGTGTCTTTTCATTG
CATGCGCCTGTTATTGCAACAACACCTGCATCCAAATAA

>HsLDC-X2

ATGACGTTTGGTCGCATGGAAGCAATTGCTCCTTCCCTGGGCATGGATGAAGCCGTTGCAGCTCGTAGCACT
TTCTTTCAATATTCCACGGATGGAGATCATGACAAGTACAGCAGCCACAGCATGAATATAGCTCTAGGTGATT
TGATTAGAAAGTTAGTGGAGTCCTCTGTCCACCACACTGTCAACATCATCCCATCTTCATCTCAGAGAGACGT
TGACATTTTCATCAATAGAACGGTCAATGAAGACAAGCTGCAGGAACCTTTCTTCGTTCTAGACCTCGGCGT
GCTTGTCCGACTGTTCTACGACTGGATCAAGGCTCTTCCAAGGGTGCTGCCTTACTACGCAGTGAAATGCAA
TCCGAGTAGACCATTGCTGGCCACCATGGCAGCCCTGGGAGCAGGCTTCGATTGTGCCAGCAAGCTGGAA
GTAGAAGAAGTGCTTGCTCTTGGAGTGGCTCCACAGAGAATCATATTCGCAAATCCATGCAAAATGCCTTCT
CACATCAATTATGCTGCATGTAAGGGTGTGCATGTCACCACCTTCGATTCTGAAGGAGAAGTGATGAAGGTG
AAGGCCAATGATCCGAATGCAACTTTACTTCTGCGTCTTAAGACGGACGACGAGAGCTCTAAATGCCCACTG
GGGGTGAAGTATGGAGCACTAATGAGCGAGGTGGATCATCTGCTGCTCGTAGCAACCAATTCTGAGGTTCC
TGTGATTGGAGTCTCCTTTCATATCGGCAGTGGAGCTTGCGATGCCCAAGCTTACACACACGCCATAGCTGC
AGCTCGATGCGTATTTGATAAAGGAGTGCAGCTAGGCCTGCCACCAATGTCTGTTCTGGACATAGGGGGAG
GCTTCACTGTGGGCACAAATGGAGAGCTGAAGCTGGCGCAAGCTGCAGAAGCAATCAACGCAGCCCTGGA
TCAGTACTTCCCACCTGGAATGGGCGTTTCCATCGTTGCAGAGCCAGGACGATTCTTTGCAGAATGCCCTAC
CACGCTAGCTACCACCATCTATGGTAAGCGAGTCCGCGGAGAATTAGTAAGAGAATATTGGATCAACGACGG
CATCTATGGCTCTATGAACAATCTCATTCATGACTACGCTGTTCTGAGGCCCCGTCCTTTGGCTTGCGCTTCCC
ATCAAACCAATCTCTCCTGCACTGGCCTTATCACCTTTTCCTCTACAGTCTTTGGACCAACCTGCGATTCTTTG

GATACTGTGCTGAAAGATTATGAGCTACCTGATCTTGCAGACGGAGACTGGATTGTGTTTTCCAACATGGGA
GCATACACCCATTGCGCTGGATCCAGCTTCAATGGCTACTCCACTTCTGATATCAGAACATACTGTGTCTTTTC
ATTGCATGCGCCTGTTATTGCAACAACACCTGCATCCAAATAA

>HsLDC-X3

ATGACGTTTGGTCGCATGGAAGCAATTGCTCCTTCCCTGGGCATGGATGAAGCCGTTGCAGCTCGTAGCACT
TTCTTTCAATATTCCACGGATGGAGATCATGACAAGTACAGCAGCCACAGCATGAATATAGCTCTAGGTGATT
TGATTAGAAAGTTAGTGGAGTCCTCTGTCCACCACACTGTCAACATCATCCCATCTTCATCTCAGAGAGACGT
TGACATTTTCATCAATAGAACGGTCAATGAAGACAAGCTGCAGGAACCTTTCTTCGTTCTAGACCTCGGCGT
GCTTGTCCGACTGTTCTACGACTGGATCAAGGCTCTTCCAAGGGTGCTGCCTTACTACGCAGTGAAATGCAA
TCCGAGTAGACCATTGCTGGCCACCATGGCAGCCCTGGGAGCAGGCTTCGATTGTGCCAGCAAGCTGGAA
GTAGAAGAAGTGCTTGCTCTTGGAGTGGCTCCACAGAGAATCATATTCGCAAATCCATGCAAAATGCCTTCT
CACATCAATTATGCTGCATGTAAGGGTGTGCATGTCACCACCTTCGATTCTGAAGGAGAAGTGATGAAGGTG
AAGGCCAATGATCCGAATGCAACTTTACTTCTGCGTCTTAAGACGGACGACGAGAGCTCTAAATGCCCACTG
GGGGTGAAGTATGGAGCACTAATGAGCGAGGTGGATCATCTGCTGCTCGTAGCAACCAATTCTGAGGTTCC
TGTGATTGGAGTCTCCTTTCATATCGGCAGTGGAGCTTGCGATGCCCAAGCTTACACACACGCCATAGCTGC
AGCTCGATGCGTATTTGATAAAGGAGTGCAGCTAGGCCTGCCACCAATGTCTGTTCTGGACATAGGGGGAG
GCTTCACTGTGGGCACAAATGGAGAGCTGAAGCTGGCGCAAGCTGCAGAAGCAATCAACGCAGCCCTGGA
TCAGTACTTCCCACCTGGAATGGGCGTTTCCATCGTTGCAGAGCCAGGACGATTCTTTGCAGAATGCCCTAC
CACGCTAGCTACCACCATCTATGGTAAGCGAGTCCGCGGAGAATTAGTAAGAGAATATTGGATCAACGACGG
CATCTATGGCTCTATGAACAATCTCATTCATGACTACGCTGTTCTGAGGCCCCGTCCTTTGGCTTGCGCTTCCC
ATCAAACCAATCTCTCCTGCACTGGCCTTATCACCTTTTCCTCTACAGTCTTTGGACCAACCTGCGATTCTTTG
GATACTGTGCTGAAAGATTATGAGCTACCTGATCTTGCAGACGGAGACTGGATTGTGTTTTCCAACATGGGA
GCATACACCCATTGCGCTGGATCCAGCTTCAATGGCTACTCCACTTCTGATATCAGAACATACTGTGTCTTTTC
ATTGCATGCGCCTGTTATTGCAAAAACACCTGCATCCAAATAA

>HsLDC-X4

ATGACGTTTGGTCGCATGGAAGCAATTGCTCCTTCCCTGGGCATGGATGAAGTCGTTGCAGCTCGTAGCACT
TTCTTTCAATATTCCACGGATGGAGATGACAACAGCAGCCTGCACAGCATGAATAAAGCTCTAGGTGATTTG
ATTAGAAAGTTAGTGGAGTCCTCAGTCCACCACACTGTCAGCATCATCCCATCTTCATCTCAGAGAGACGTTG
ACATTTTCATCAATAGCACGGTCAATGAAGACAAGCTGCAGGAACCTTTCTTCGTTCTAGACCTCGGCGTGC
TTGTTCGACTGTTCTACGATTGGATCAAGGCTCTTCCAAGGGTGCTGCCTTACTACGCAGTGAAATGCAATCC
GAGTAGACCATTGCTGGCCACCATGGCAGCCCTGGGAGCAGGCTTCGATTGTGCCAGCAAGCTGGAAGTA
GAAGAAGTGCTTGCTCTTGGAGTGGCTCCACAGAGAATCATATTCGCAAATCCATGCAAAATGCCTTCTCAC
ATCAATTACGCTGCATGTAAGGGTGTGCATGTCACCACCTTCGATTCTGAAGGAGAAGTGATGAAGGTGAAG
GCCAATGATCCGAATGCAACTTTACTTCTGCGTCTTAAGACGGACGATGAGAGCTCCAAATGCCCACTGGGG
GTGAAGTATGGGGCACTAATGAGCGAGGTGGATCATCTGCTGCTCGTAGCAGCCAATGCTGAGGTTCCTGT
GATTGGAGTCTCCTTTCATATCGGCAGTGGAGCTTGCGATGCCCAAGCTTACACACACGCCATAGCTGCAGC
TCGAAGCGTATTTGATAAAGGAGAGCAGCTAGGCCTGCCACCAATGTCTGTTCTGGACATAGGGGGAGGCT
TCACTGTGGGCACAAATGGAGAGCTGAAGCTGGCGGAAGCTGCAGAAGCAATCAACGCAGCCCTGGATCA
GTACTTCCCACCTGGAATGGGCGTTTCCATCGTTGCAGAGCCAGGAAGATTCTTTGCAGAATGCCCTACCAC
GCTAGCTACCACCATCTATGGTAAGCGAGTCCGCGGAGAATTAGTAAGAGAATATTGGATCAACGACGGCAT
CTATGGCTCTATGAACAATCTCATTCATGACTACGCTGTTCTGAGGCCCCGTCCTTTGGCTTGCGCTTCCCATC
AAACCAATCTCTCCTGCACTGGCCTTATCACCTTTTCCTCTACAGTCTTTGGACCAACCTGCGATTCTTTGGAT

ACTGTGCTGAAAGATTATGAGCTACCTGATCTTGCAGACGGAGATTGGATTGTGTTTTCCAACATGGGAGCA
TACACCCATTGCGCTGGATCCAGCTTCAATGGCTACTCCACTTCTGATATCAGAACATACTGTGTCTTTTCATT
GCATGCGCCTGTTATTGCAACAACACCTGCATCCAAATAA

>HsLDC-X5

ATGACGTTTGGTCGCATGGAAGCAATTGCTCCTTCCCTGGGCATGGATGAAGCCGTTGCAGCTCGTAGCACT
TTCTTTCAATATTCCACGGATGGAGATCATGACAAGTACAGCAGCCACAGCATGAATATAGCTCTAGGTGATT
TGATTAGAAAGTTAGTGGAGTCCTCTGTCCACCACACTGTCAACATCATCCCATCTTCATCTCAGAGAGACGT
TGACATTTTCATCAATAGAACGGTCAATGAAGACAAGCTGCAGGAACCTTTCTTCGTTCTAGACCTCGGCGT
GCTTGTCCGACTGTTCTACGACTGGATCAAGGCTCTTCCAAGGGTGCTGCCTTACTACGCAGTGAAATGCAA
TCCGAGTAGACCATTGCTGGCCACCATGGCAGCCCTGGGAGCAGGCTTCGATTGTGCCAGCAAGCTGGAA
GTAGAAGAAGTGCTTGCTCTTGGAGTGGCTCCACAGAGAATCATATTCGCAAATCCATGCAAAATGCCTTCT
CACATCAATTACGCTGCATGTAAGGGTGTGCATGTCACCACCTTCGATTCTGAAGGAGAAGTGATGAAGGTG
AAGGCCAATGATCCGAATGCAACTTTACTTCTGCGTCTTAAGACGGACGATGAGAGCTCTAAATGCCCACTG
GGGGTGAAGTATGGAGCACTAATGAGCGAGGTGGATCATCTGCTACTCGTAGCAGCCAATGCTGAGGTTCC
TGTGATTGGAGTCTCCTTTCATATCGGAAGTGGAGCTTGCGATGCCCAAGCTTACACACACGCCATAGCTGC
AGCTCGAAGCGTATTTGATAAAGGAGTGCAGCTAGGCCTGCCACCAATGTCTGTTCTGGACATAGGGGGAG
GCTTCACTGTGGGCACAAATGGAGAGCTGAAGCTGGCGGAAGCTGCAGAAGCAATCAACGCAGCCCTGGA
TCAGTACTTCCCACCTGGAATGGGCGTTTCCATCGTTGCAGAGCCAGGAAGACTCTTTGCAGAATGCCCTAC
CACGCTAGCTACCACCATCTATGGTAAGCGAGTCCGCGGAGAATTAGTAAGAGAATATTGGATCAACGACGG
CATCTATGGCTCTATGAACAATCTCATTCATGACTACGCTGTTCTGAGGCCCCGTCCTTTGGCTTGCGCTTCCC
ATCAAACCAATCTCTCCTGCACTGGCCTTATCACCTTTTCCTCTACAGTCTTTGGACCAACCTGCGATTCTTTG
GATACTGTGCTGAAAGATTATGAGCTACCTGATCTTGCAGACGGAGACTGGATTGTGTTTTCCAACATGGGA
GCATACACCCATTGCGCTGGATCCAGCTTCAATGGCTACTCCACTTCTGATATCAGAACATACTGTGTCTTTTC
ATTGCATGCGCCTGTTATTGCAACAACACCTGCATCCAAATAA

>HsLDC-X6

ATGACGTTTGGTCGCATGGAAGCAATTGCTCCTTCCCTGGGCATGGATGAAGCCGTTGCAGCTCGTAGCACT
TTCTTTCAATATTCCACGGATGGAGATCATGACAAGTACAGCAGCCACAGCATGAATATAGCTCTAGGTGATT
TGATTAGAAAGTTAGTGGAGTCCTCTGTCCACCACACTGTCAACATCATCCCATCTTCATCTCAGAGAGACGT
TGACATTTTCATCAATAGAACGGTCAATGAAGACAAGCTGCAGGAACCTTTCTTCGTTCTAGACCTCGGCGT
GCTTGTCCGACTGTTCTACGACTGGATCAAGGCTCTTCCAAGGGTGCTGCCTTACTACGCAGTGAAATGCAA
TCCGAGTAGACCATTGCTGGCCACCATGGCAGCCCTGGGAGCAGGCTTCGATTGTGCCAGCAAGCTGGAA
GTAGAAGAAGTGCTTGCTCTTGGAGTGGCTCCACAGAGAATCATATTCGCAAATCCATGCAAAATGCCTTCT
CACATCAATTATGCTGCATGTAAGGGTGTGCATGTCACCACCTTCGATTCTGAAGGAGAAGTGATGAAGGTG
AAGGCCAATGATCCGAATGCAACTTTACTTCTGCGTCTTAAGACGGACGACGAGAGCTCTAAATGCCCACTG
GGGGTGAAGTATGGAGCACTAATGAGCGAGGTGGATCATCTGCTGCTCGTAGCAACCAATTCTGAGGTTCC
TGTGATTGGAGTCTCCTTTCATATCGGCAGTGGAGCTTGCGATGCCCAAGCTTACACACACGCCATAGCTGC
AGCTCGATGCGTATTTGATAAAGGAGTGCAGCTAGGCCTGCCACCAATGTCTGTTCTGGACATAGGGGGAG
GCTTCACTGTGGGCACAAATGGAGAGCTGAAGCTGGCGCAAGCTGCAGAAGCAATCAACGCAGCCCTGGA
TCAGTACTTCCCACCTGGAATGGGCGTTTCCATCGTTGCAGAGCCAGGACGATTCTTTGCAGAATGCCCTAC
CACGCTAGCTACCACCATCTATGGTAAGCGAGTCCGCGGAGAATTAGTAAGAGAATATTGGATCAACGACGG
CATCTATGGCTCTATGAACAATCTCATTCATGACTACGCTGTTCTGAGGCCCCGTCCTTTGGCTTGCGCTTCCC
ATCAAACCAATCTCTCCTGCACTGGCCTTATCACCTTTTCCTCTACAGTCTTTGGACCAACCTGCGATTCTTTG

GATACTGTGCTGAAAGATTATGAGCTACCTGATCTTGCAGACGGAGACTGGATTGTGTTTTCCAACATGGGA
GCATACACCCATTGCGCTGGATCCAGCTTCAATGGCTACTCCACTTCTGATATCAGAACATACTGTGTCTTTTC
ATTGCATGCGCCTGTTATTGCAACAACACCTGCATCCAAATAA

>AB915697.1
ATGGCATCTTGTAACTCTACAATGATGTATAGCTTGGATGCTTTTCTTGGGTTGGAGGATGCAGATTACT
GTGAATTTTCTTGCTCTGGGAGGAAAACCAAGTGTGCCAAGTCTTCTGCATTTGAGCCACGTGAAGTAGA
TAGCATGCCTCTCACTACTGCTCTCAACAAGGTTCTAGAATCCGCCTGTAATGGTCTCAGCAAAGTACCC
ATCATCACATCTAGCTCCCAGCAAGATATAGATGCTTTCATAGAGAGCACCATCCAATCCAATAATCTCC
AAGATGCTTTCTATGTTCTAGATCTAGCTGTAGTTGTCCAACTATTCAATGACTGGGTTACTGCTCTACC
CAAAGTTAAGCCATACTATGCAGTGAAGTGCAACCCCTCTCCTTCTCTTTTGTCCACATTGGCTGCTCTT
GGAGCTGGATTTGACTGCGCAAGCCAGTCTGAGCTGGAGCTGGTAACATCAATGGGTGTATCAGCCCAGA
GAATCATATTTGCTAATCCGTGCAAGATGCCATCACACATCAAGTATGCAACGTCTGTGGGAGTGAACCT
CACTACCTATGACTGTGAACACGAGGTGAGGAAGGTGAAGGTGCACAGTCCCAACGCCAAGCTGCTGCTG
AGGCTTCGAGCCGATGACAGGAACTCAAAGTGGCCTCTGGGGGTCAAGTATGGAGCTCTCTCTTCTGAAG
TGGAGCACTTGCTCATGGCTGCTGCCAATGCGCAGGTAGACGTGGTGGGTGTGTCCTTTCACATTGGCAG
TGGCGCTTCCGATGCTCGTTCGTATCTGGATGCCATTGCTGCTGCTCGAGGAGTGTTCGAGAAGGCCCTG
AGCATGGGCCTCCCGGCAATGCACATTCTCGACATTGGAGGAGGCTTTACTGTGGGCAACAACGGAGAGC
TAAAGCTGGCAGAAGCAGCGAAGGTGATCAACGCTGCTCTTGAGCAGTACTTCCCAGAGGAGCTGGGGGT
GAGCATCATCGCGGAGCCTGGCCGGTACTTCGCGGAGGCGCCCACCACGCTTGCAGCTATGATCTACGGC
AAGAGAGTGCGAGAGGAGGTGAGGGAGTATTGGATAAACGACGGCATCTACGGCACTATGAACTGCCTCA
TCCATGACTACGCAGTCCTCTGTCCTCGACCTCTGGCTTGTGCTTCTCAGAGGAGCAACGTCTCCTGCGC
GAAGCTCCCTTTGCACAAGTCCACTGTGTTTGGTCCCACCTGTGATTCCCTCGACACTGTCCTGAAGGAG
CATCCCTTGCCAGATCTAGTGGATGGAGACTGGATCGTTTTTCCTAACATGGGAGCCTACACTCACTGTG
CTGGATCTAGCTTCAACGGCTTCGACACCTCCGCTATTCCAACGTATTGCGTCTTCTCGCTGAATGCCAG
AGGCTCGTCTGTAGCTCTAGACACTCTGAAGACGAGCTTTCACACAGAGTGTGATTTTTGTGACAAGCTA
AGTAGCACCAGTACTGAAGCCAGTACTGAAGAAAGCAGCCCTGGCGAAGACAGCGAGTAA

>AB915696.1
ATGGCATCTTGTAACTCTACAGTGATGTATAGCTTGGATGCTTTTCTTGGGTTGGAGAATGTAGATTACT
GTGAATTTTCTTGCTCTGGGAGGAAAACCAAGTGTGCCAAGTCTTCTGCATTTGAGCCACGTGAAGTAGG
TAGCATGCCTCTCACTACTGCTCTCAACAAGGTTCTAGAATCCGCCTGCAATGGTCTCATGATCAGCAAA
GTACCCATCATCACATCTAGCTCCCAGCAAGATATAGATGCTTTCATAGAGAGCACCATCCAATCCAATA
ATCTCCAAGATGCTTTCTATGTTCTAGATCTAGCTGTAGTTGTCCAACTATTCAATGACTGGGTTGCTGC
TCTTCCGAAAGTTAAGCCATACTATGCAGTGAAGTGCAACCCCTCTCCTTCTCTTTTGTCCACATTGGCT
GCTCTTGGAGCTGGATTCGACTGCGCAAGCCAGTCTGAGCTGGAGCTGGTAACATCAATGGGTGTATCAG
CCCAGAGAATCATATTTGCTAATCCGTGCAAGATGCCATCACACATCAAGTATGCAACGTCTGTGGGAGT
GAACCTCACTACCTATGACTGTGAACACGAGGTGAGGAAGGTGAAGGTGCACAGTCCCAACGCCAAGCTG
CTGCTGAGGCTTCGAGCTGATGACAGGAGCTCAAAGTGGCCTCTGGGTGTCAAGTATGGAGCTCTCTCTT
CTGAAGTGGAGCACTTGCTCATGGCTGCTGCCAATGCGCAGTTAGACGTGGTGGGTGTGTCCTTTCACAT
TGGCAGTGGCGCTTCCGATGCTCGTTCGTATCTGGATGCCATTGCTGCTGCTCGAGGAGTGTTCGAGAAG
GCCCTGAGCATGGGCCTCCCGGCAATGCACATTCTCGACATTGGAGGAGGCTTTACTGTGGGCAACAACG
GAGAGCTAAAGCTGGCAGAAGCAGCGAAGGTGATCAACGCTGCTCTTGAGCAGTACTTCCCAGAGGAGCT
GGGGGTGAGCATCATCGCGGAGCCTGGCCGGTACTTCGCGGAGGCGCCCACCACGCTTGCAGCTATGATC

TACGGCAAGAGAGTGCGACAGGAGGTGAGGGAGTATTGGATAAACGACGGCATCTACGGCACTATGAACT
GCCTCATCCATGACTACGCAGTCCTCTGTCCTCGACCTCTGGCTTGTGCTTCTCAGAGGAGCAACCTCTC
CTGCGCGAAGCTCCCTTTGCACAAGTCCACTGTGTTTGGTCCCACCTGTGATTCCCTCGACACTGTCCTG
AAGGAGCATCCCTTGCCAGATCTAGTGGATGGAGACTGGATCGTCTTTCCTAACATGGGAGCCTACACTC
ACTGTGCTGGATCTAGCTTCAACGGCTTCGACACCTCCGCTATTCCAACGTATTGCGTCTTCTCGCTGAA
TGCCAGAGGCTCGTCTGTAGCTCTAGACACTCTGAAGACGAGCTTTCACACAGAGTGTGATATTTGTGAC
AAGCTAAGTAGCACCAGTACTGAAGAAAGCAGCCCTGGCGAAGACAGCGAGTAA

>Unigene94617
ATGCCTCTCACTACTGCTCTCAACAAGGTTCTAGAATCCGCCTGTAATGGTCTCAGCAAA
GTACCCATCATCACATCTAGCTCCCAGCAAGATATAGATGCTTTCATAGAGAGCACCATC
CAATCCAATAATCTCCAAGATGCTTTCTATGTTCTAGATCTAGCTGTAGTTGTCCAACTA
TTCAATGACTGGGTTACTGCTCTACCCAAAGTTAAGCCATACTATGCAGTGAAGTGCAAC
CCCTCTCCTTCTCTTTTGTCCACATTGGCTGCTCTTGAAGCTGGATTTGACTGCGCAAGC
CAGTCTGAGCTGGAGCTGGTAACATCAATGGGTGTATCAGCCCAGAGAATCATATTTGCT
AATCCGTGCAAGATGCCATCACACATCAAGTATGCAACGTCTGTGGGAGTGAACCTCACT
ACCTATGACTGTGAACACGAGGTGAGGAAGGTGAAGGTGCACAGTCCCAACGCCAAGCTG
CTGCTGAGGCTTCGAGCCGATGACAGGAACTCAAAGTGGCCTCTGGGTGTCAAGTATGGA
GCTCTCTCTTCTGAAGTGGAGCACTTGCTCATGGCTGCTGCCAATGCGCAGGTAGACGTG
GTGGGTGTGTCCTTTCACATTGGCAGTGGCGCTTCCGATGCTCGTTCGTATCTGGATGCC
ATTGCTGCTGCTCGAGGAGTGTTCGAGAAGGCCCTGAGCATGGGCCTCCCGGCAATGCAC
ATTCTCGACATTGGAGGAGGCTTTACTGTGGGCAACAACGGAGAGCTAAAGCTGGCAGAA
GCAGCGAAGGTGATCAACGGTGCTCTTGAGCAGTACTTCCCAGAGGAGCTGGGGGTGAGC
ATCATCGCGGAGCCTGGCCGGTACTTCGCGGAGGCGCCCACCACGCTTGCAGCTATGATC
TACGGCAAGAGAGTGCGAGAGGAGGTGAGGGAGTATTGGATAAACGACGGCATCTACGGC
ACTATGAACTGCCTCATCCATGACTACGCAGTCCTCTGTCCTCGACCTCTGGCTTGTGCT
TCTCAGAGGAGCAACGGCCAACTCTCCTGCGCGAAGCTCCCTTTGCACAAGTCCACTGTG
TTTGGTCCCACCTGTGATTCCCTCGACACTGTCCTGAAGGAGCATCCCTTGCCAGATCTA
GTGGATGGAGACTGGATCGTTTTTCCTAACATGGGAGCCTACACTCACTGTGCTGGATCT
AGCTTCAACGGCTTCGACACCTCCGCTATTCCAACGTATTGCGTCTTCTCGCTGAATGCC
AGAGGCTCGTCTGTAGCTCTAGACACTCTGAAGACGAGCTTTCACACAGAGTGTGATTTT
TGTGACAAGCTAAGTAGCACCAGTACTGAAGCCAGTACTGAAGAAAGCAGCCCTGGCGAA
GACAGCGAGTAA

>Unigene94988
ATGACGTTTGGTCGCATGGAAGCAATTGCTCCTTCCCTGGGCATGGATGAAGTCGTTGCA
GCTCGTAGCACTTTCTTTCAATATTCCACGGATGGAGATCATGACAAGTACAGCAGCCAC
AGCATGAATATAGCTCTAGGTGATTTGATTAGAAAGTTAGTGGAGTCCTCTGTCCACCAC
ACTGTCAACATCATCCCATCTTCATCTCAGAGAGACGTTGACATTTTCATCAATAGCACG
GTCAATGAAGACAAGCTGCAGGAACCTTTCTTCGTTCTAGACCTCGGCGTGCTTGTTCGA
CTGTTCTACGATTGGATCAAGGCTCTTCCAAGGGTGCTGCCTTACTACGCAGTGAAATGC
AATCCGAGTAGACCATTGCTGGCCACCATGGCAGCCCTGGGAGCAGGCTTCGATTGTGCC
AGCAAGCTGGAAGTAGAAGAAGTGCTTGCTCTTGGAGTGGCTCCACAGAGAATCATATTC
GCAAATCCATGCAAAATGCCTTCTCACATCAATTACGCTGCATGTAAGGGTGTGCATGTC

ACCACCTTCGATTCTGAAGGAGAAGTGATGAAGGTGAAGGCCAATGATCCGAATGCAACT
TTACTTCTGCGTCTTAAGACGGACGATGAGAGCTCCAAATGCCCACTGGGGGTGAAGTAT
GGGGCACTAATGAGCGAGGTGGATCATCTGCTGCTCGTAGCAGCCAATGCTGAGGTTCCT
GTGATTGGAGTCTCCTTTCATATCGGCAGTGGAGCTTGCGATGCCCAAGCTTACACACAC
GCCATAGCTGCAGCTCGAAGCGTATTTGATAAAGGAGAGCAGCTAGGCCTGCCACCAATG
TCTGTTCTGGACATAGGGGGAGGCTTCACTGTGGGCACAAATGGAGAGCTGAAGCTGGCG
GAAGCTGCAGAAGCAATCAACGCAGCCCTGGATCAGTACTTCCCACCTGGAATGGGCGTT
TCCATCGTTGCAGAGCCAGGAAGATTCTTTGCAGAATGCCCTACCACGCTAGCTACCACC
ATCTATGGTAAGCGAGTCCGCGGAGAATTAGTAAGAGAATATTGGATCAACGACGGCATC
TATGGCTCTATGAACAATCTCATTCATGACTACGCTGTTCTGAGGCCCCGTCCTTTGGCT
TGCGCTTCCCATCAAACCAATCTCTCCTGCACTGGCCTTATCACCTTTTCCTCTACAGTC
TTTGGACCAACCTGCGATTCTTTGGATACTGTGCTGAAAGATTATGAGCTACCTGATCTT
GCAGACGGAGATTGGATTGTGTTTTCCAACATGGGAGCATACACCCATTGCGCTGGATCC
AGCTTCAATGGCTACTCCACTTCTGATATCAGAACATACTGTGTCTTTTCATTGCATGCG
CCTGTTATTGCAACAACACCTGCATCCAAATAA

2) Copper amine oxidases (CAO)

>HsCAO
ATGAGCCAATCCCCAGACGTAATAGAACAGCAACACACAAATAGGGTGCCAAAAGACAAGCAGCCATTCC
CTTCTGCCCAACACCGACACCCATTGGATCCTTTGAGGTGGGATGAAATCCTCCTTGCACGCTCTGTTCT
GCTCAATTCGTCCTTGCTTACAGAGGGCCATCCAACAGTACACATCATCACTCTAGAAACACCAGAGAAG
GAGGAAGTACTCAACTGGAAGCCTGGCCAATTACCTGCTCCACTTAGAAGAGCTTATGTTGAAACGATTC
TGTTTGGGAAAACTCACAAAATCGTGGTGGATGTAGCCGCAGGAGCTTTGGTTTCTGATGAAATCCACCA
TGCCCCAGGCTACCCCTCACTTTCTTCAAATGATATCTTAATCGTAGCTACCCTCCCTTCTACCCACCCC
CCATTTCTGCAATCCTTAAAGGCCAGAGGTGTGGGAGTTTCAGATGTCGTCTGCTTGCCAATCTCTCCAG
GATGGTTTGGCATACCTGAGGAGGAAGGCAAGAGGCTTGTCAAAGCTCTTTGCTACAACAAAAATGGATC
TGCGAATGTTTTCATGAGGCCACTGGAGGGGATTGTCATTCTCCTGGATTTGGACAGAAAACAAATCCTC
AAGTACGTTGATGACAGAAAAGTCCCAATTCCCAAAGTAGAAGGCACAGACTACAGGCTTTTTGCTCAGA
AGCCTCCACTCATGAAGCCATTGAATCCCATATCCTTGGAGCAACCTCTTGGTCCTAGCTTCAAAGTGGA
GGGCAACTTGGTGAAGTGGGCAAACTGGGAATTTCATGTCCGGCCTGATTTTAGAGCTGGGATGGTGATC
TCTCAGGCTGTCATCCATGATCCAGAGACAGGTGAAGCTAGGAGTGTCCTGTACGAGGGGTTTCCTTCCG
AGCTCTTTGTCCCCTACATGGATCCTTCAGAAGGATGGTATTTCAAAACATACATGGATTCTGGAGAGTA
TGGATTAGGAATGCTGGCCTTACCTCTGCAACCTTTAAATGATTGCCCAAGAAATGCACACTACTTTGAT
GCTGTCTTTGCAGGGCCTGATGGCAATCCCTATGTGACACCAAACATTCTGTGTGTGTTCGAGCGTTATG
CTGGAGACGTCGCATGGAGACACTCAGAGGCCTTTGTCCAAGATTTTGAGGTTCATGAGGTGCGACCAAA
AGTTACACTCGTGGTCCGAATGGTTGGATCAGTGGGGAATTATGACTACATTTTTGACTGGGAGTTTCAG
ACGGATGGCATTCTCCGAGTGAATGTTGGAATGACTGGCTTGTTAATGGTAAAGGCCACCTCTATCAACT
CCATTGCGGAGAATATTGTTGACTTGCATGGAACATTGGTTTCCGAGAATACAATAGGTGTTTTCCATGA
CCATTTTATCAACTTCCATCTGGATCTTGATATCGACGGGTTGACAAATACTTTCATTAAGAAGATACTT
AAACGAAAAAATGTTGTCAACAATGAATCACCAAGAAAGAGCTACTGGACAACTGAAAATCAAATAGCAG
AAACTGAGGACGACGCCAAAATCAGATTGAAAGCATTTGAGCCTTCTGAATTTCATATTGTGAACTCAAA
AAAGAGGACTAGGCTCGGGAATCCTGTAGGGTATCGGATTGTTCCTGGATTCACTGCAGACAGTTTACTT
TCATCAGTTGATCCACCTCAACACCGCGCAGCCTTCATTGATAATCAGATTTGGGTAACTCCATTGAACA

AGAGTGAACGATGGGCTGGTGGGCTGTTTGTCTATGAGAGCCACGGCGAGGATACTCTTGCTGTTTGGTC
CAAGAGGAATAGAGCGATCCAAGGAAGAGATATAGTGCTGTGGTACACAATGGGCTTCCATCACGTTCCA
TGTCAGGAGGATTTTCCAATAATGCCCACATTAAGCGGCAGCTTTGAGCTGAAGCCAAGTAATTTCCTTG
AGAGAAATCCTATACTAAAAACACTGCCAAATTACCCTTCCCAGCTGCCCAAGTGTTCAATTGGCAGCCA
TGTTGACGAGAGGTAG

>CL4248.1
ATGAGTAGTTTCCATGGTGGGCATTTGGTGTGCTTCTCTGGGTTTTGTATCCTTCTGCTT
GGTGTTGCCAATCTATGGTTCTGCATTCCAGCAGGATTCTCTGAATGCTCCATTCCAACA
TTTCCATGGAATATTACTGTCACAAGATTTCTTCCCAGCAGCCAATCCCCAGACGTAATA
GAACAGCAACACACAAATAGGGTGCCAAAAGACAAGCAGCCATTCCCTTCTGCCCAACAC
CGACACCCATTGGATCCTTTGACGTGGGATGAAATCCTCCTTGCACGCTCTGTTCTGCTC
AATTCGTCCTTGCTTACAGAGGGCCATCCAACAGTACACATCATCACTCTAGAAACACCA
GAGAAGGAGGAAGTACTCAACTGGAAGCCTGGCCAATTACCTGCTCCACTTAGAAGAGCT
TATGTTGAAACGATTCTGTTTGGGAAAACTCACAAAATCGTGGTGGATGTAGCCGCAGGA
GCTTTGGTTTCTGATGAAATCCACCATGCCCCAGGCTACCCCTCACTTTCTTCAAATGAT
ATCTTAATCGTAGCTACCCTCCCTTCTACCCACCCCCCATTTCTGCAATCCTTAAAGGCC
AGAGGTGTGGGAGTTTCAGATGTCGTCTGCTTGCCAATCTCTCCAGGATGGTTTGGCATA
CCTGAGGAGGAAGGCAAGAGGCTTGTCAAAGCTCTTTGCTACAACAAAAATGGATCTGCG
AATGTTTTCATGAGGCCACTGGAGGGGATTGTCATTCTCCTGGATTTGGACAGAAAACAA
ATCCTCAAGTACGTTGATGACAGAAAAGTCCCAATTCCCAAAGTAGAAGGCACAGACTAC
AGGCTTTTTGCTCAGAAGCCTCCACTCATGAAGCCATTGAATCCCATATCCTTGGAGCAA
CCTCTTGGTCCTAGCTTCAAAGTGGAGGGCAACTTGGTGAAGTGGGCAAACTGGGAATTT
CATGTCCGGCCTGATTTTAGAGCTGGGATGGTGATCTCTCAGGCTGTCATCCATGATCCA
GAGACAGGTGAAGCTAGGAGTGTCCTGTACGAGGGGTTTCCTTCCGAGCTCTTTGTCCCC
TACATGGATCCTTCAGAAGGATGGTATTTCAAAACATACATGGATTCTGGAGAGTATGGA
TTAGGAATGCTGGCCTTACCTCTGCAACCTTTAAATGATTGCCCAAGAAATGCACACTAC
TTTGATGCTGTCTTTGCAGGGCCTGATGGCAATCCCTATGTGACACCAAACATTCTGTGT
GTGTTCGAGCGTTATGCTGGAGACGTCGCATGGAGACACTCAGAGGCCTTTGTCCAAGAT
TTTGAGGTTCATGAGGTGCGACCAAAAGTTACACTCGTGGTCCGAATGGTTGGATCAGTG
GGGAATTATGACTACATTTTTGACTGGGAGTTTCAGACGGATGGCATTCTCCGAGTGAAT
GTTGGAATGACTGGCTTGTTAATGGTAAAGGCCACCTCTATCAACTCCATTGCGGAGAAT
ATTGTTGACTTGCATGGAACATTGGTTTCCGAGAATACAATAGGTGTTTTCCATGACCAT
TTTATCAACTTCCATCTGGATCTTGATATCGACGGGTTGACAAATACTTTCATTAAGAAG
ATACTTAAACGAAAAAAATGTTGTCAACAATAG

>CL4248.2
ATGAGTAGTTTCCATGGTGGGCATTTGGTGTGCTTCTCTGGGTTTTGTATCCTTCTGCTT
GGTGTTGCCAATCTATGGTTCTGCATTCCAGCAGGATTCTCTCAATGCTCCATTCCAGCA
TTTCCATGGAATATTACTGTCACAAGATTTCTTCCCAGCACCCAATCCCCAGGCCTCAGC
AAAGAACAGCAGCACACAAATAGGGTGCCAAAAGACAAGCAGCCATTCCCCTCTGCCCAA
CATCGACACCCATTGGATCCTTTAACGTGGGATGAAATCCTCCTTGCACGCTCTGTTCTG
CTCAATTCGTCCTTGCTTACAGAGGGCCATCCAACAGTACACATCATCACTCTAGAAACA
CCAGAGAAGGAGGAAGTACTCAACTGGAAGGCTGGCAAATCACCTGCTCCACCTAGAAGA

GCTTATGTTGAAACGATTCTATTTGGAGAAACTCACAAAATTGTGGTGGATGTAGCCGTA
GGATCTCTGGTTTCTGATGAAATCCACCATGCCCCAGGCTACCCCTCAATTTCTTCGGAT
GATGTCTCAATCACAGCTACCCTCCCTTCTACCTACCCCCCATTTCTGCAATCCTTGCAG
GCCAGAGGTCTGGGAGTGTCAGATGTCGTCTGCTTGCCAATCTCTCCAGGATGGTTTGGC
ATACCTGAGGAGGAAGGCAAGAGGCTTGTCAAAGCTCTTTGCTACAACAAAAATGGATCT
GCGAATGTTTTCATGAGGCCACTGGAGGGGATTGTCATTCTCCTGGATTTGGACAGAAAA
CAAATCCTCAAGTACGTTGATGACAGAAAAGTCCCAATTCCCAAAGTAGAAGGCACAGAC
TACAGGCTTTTTGCTCAGAAGCCTCCACTCATGAAGCCATTGAATCCCATATCCTTGGAG
CAACCTCTTGGTCCTAGCTTCAAAGTGGAGGGCAACTTGGTGAAGTGGGCAAACTGGGAA
TTTCATGTCCGGCCTGATTTTAGAGCTGGGATGGTGATCTCTCAGGCTGTCATCCATGAT
CCAGAGACAGGTGAAGCTAGGAGTGTCCTGTACGAGGGGTTTCCTTCCGAGCTCTTTGTC
CCCTACATGGATCCTTCAGAAGGATGGTATTTCAAAACATACATGGATTCTGGAGAGTAT
GGATTAGGAATGCTGGCCTTACCTCTGCAACCTTTAAATGATTGCCCAAGAAATGCACAC
TACTTTGATGCTGTCTTTGCAGGGCCTGATGGCAATCCCTATGTGACACCAAACATTCTG
TGTGTGTTCGAGCGTTATGCTGGAGACGTCGCATGGAGACACTCAGAGGCCTTTGTCCAA
GATTTTGAGGTTCATGAGGTGCGACCAAAAGTTACACTCGTGGTCCGAATGGTTGGATCA
GTGGGGAATTATGACTACATTTTTGACTGGGAGTTTCAGACGGATGGCATTCTCCGAGTG
AATGTTGGAATGACTGGCTTGTTAATGGTAAAGGCCACCTCTATCAACTCCATTGCGGAG
AATATTGTTGACTTGCATGGAACATTGGTTTCCGAGAATACAATAGGTGTTTTCCATGAC
CATTTTATCAACTTCCATCTGGATCTTGATATCGACGGGTTGACAAATACTTTCATTAAG
AAGATACTTAAACGAAAAAATGTTGTCAACAATGAATCACCAAGAAAGAGCTACTGGACA
ACTGAAAATCAAATAGCAGAAACTGAGGACGACGCCAAAATCAGATTGAAAGCATTTGAG
CCTTCTGAATTTCATATTGTGAACTCAAAAAAGAGGACGAGGCTTGGGAATCCTGTAGGG
TATCGGATTGTTCCTGGATTCACTGCAGACAGTTTACTTTCATCAGTTGATCCACCTCAA
CACCGCGCAGCCTTCATTGATAATCAGATTTGGGTAACTCCATTGAACAAGAGTGAACGA
TGGGCTGGTGGGCTGTTTGTCTATGAGAGCCACGGCGAGGATACTCTTGCTGTTTGGTCC
AAGAGGAATAGAGCGATCCAAGGAAGAGATATAGTGCTGTGGTACACAATGGGCTTCCAT
CACGTTCCATGTCAGGAGGATTTTCCAATAATGCCCACATTAAGCGGCAGCTTTGAGCTG
AAGCCAAGTAATTTCCTTGAGAGAAATCCTATACTAAAAACACTGCCAAATTACCCTTCC
CAGCTGCCCAAGTGTTCAATTGGCAGCCATGTTGACGAGAGGTAG

>CL4248.3
ATGAGTAGTTTCCATGGTGGGCATTTGGTGTGCTTCTCTGGGTTTTGTATCCTTCTGCTT
GGTGTTGCCAATCTATGGTTCTGCATTCCAGCAGGATTCTCTCAATGCTCCATTCCAGCA
TTTCCATGGAATATTACTGTCACAAGATTTCTTCCCAGCACCCAATCCCCAGGCCTCAGC
AAAGAACAGCAGCACACAAATAGGGTGCCAAAAGACAAGCAGCCATTCCCCTCTGCCCAA
CATCGACACCCATTGGATCCTTTAACGTGGGATGAAATCCTCCTTGCACGCTCTGTTCTG
CTCAATTCGTCCTTGCTTACAGAGGGCCATCCAACAGTACACATCATCACTCTAGAAACA
CCAGAGAAGGAGGAAGTACTCAACTGGAAGGCTGGCAAATCACCTGCTCCACCTAGAAGA
GCTTATGTTGAAACGATTCTATTTGGAGAAACTCACAAAATTGTGGTGGATGTAGCCGTA
GGATCTCTGGTTTCTGATGAAATCCACCATGCCCCAGGCTACCCCTCAATTTCTTCGGAT
GATGTCTCAATCACAGCTACCCTCCCTTCTACCTACCCCCCATTTCTGCAATCCTTGCAG
GCCAGAGGTCTGGGAGTGTCAGATGTCGTCTGCTTGCCAATCTCTCCAGGATGGTTTGGC
ATACCTGAGGAGGAAGGCAAGAGGCTTGTCAAAGCTCTCTGCTACAACAAAAATGGATCT

GTGAATGTTTTCATGAGGCCATTGGAGGGGATTGTATTTCTCCTGGATTTGGACAGAAAA
CAAATCCTCAAGTACGTTGATGACAGAAAAGTCCCAATTCCCAAAGTAGAAGGCACAGAC
TACAGGCTTTTTGCTCAGAAGCCTCCACTCATGAAGCCATTGAATCCCATATCCTTGGAG
CAACCTCTTGGTCCTAGCTTCAAAGTGGAGGGCAACTTGGTGAAGTGGGCAAACTGGGAA
TTTCATGTCCGGCCTGATTTTAGAGCTGGGATGGTGATCTCTCAGGCTGTCATCCATGAT
CCAGAGACAGGTGAAGCTAGGAGTGTCCTGTACGAGGGGTTTCCTTCCGAGCTCTTTGTC
CCCTACATGGATCCTTCAGAAGGATGGTATTTCAAAACATACATGGATGCTGGAGAGTAT
GGGTTGGGACCGCTGGCCTTACCTCTGCAACCTTTGAATGATTGCCCAAGAAATGCACGC
TACTTCGATGCTGTCTTTGCAGGGTCTGACGGCAATCCCTATGTGACACCAAACATTCTG
TGTGTGTTCGAGCGTTATGCTGGAGACGTCGCATGGAGACACTCAGAGGCCTTTATCCAA
GATTTTGAGATTCATGAAGTCCGACCAAAAGTTACACTCGTGGTCCGAATGGTTGGATCA
GTGGGGAATTATGACTACATTTTTGACTGGGAGTTTCAGACGGATGGCATTCTCCGAGTG
AATGTTGGAATGACTGGCTTGTTAATGGTAAAGGCCACCTCTATCAACTCCATTGCGGAG
AATATTGTTGACTTGCATGGAACATTGGTTTCCGAGAATACAATAGGTGTTTTCCATGAC
CATTTTATCAACTTCCATCTGGATCTTGATATCGACGGGTTGACAAATACTTTCATTAAG
AAGATACTTAAACGAAAAAATGTTGTCAACAATGAATCACCAAGAAAGAGCTACTGGACA
ACTGAAAATCAAATAGCAGAAACTGAGGACGACGCCAAAATCAGATTGAAAGCATTTGAG
CCTTCTGAATTTCATATTGTGAACTCAAAAAAGAGGACTAGGCTCGGGAATCCTGTAGGG
TATCGGATTGTTCCTGGATTCACTGCAGACAGTTTACTTTCATCAGTTGATCCACCTCAA
CACCGCGCAGCCTTCATTGATAATCAGATTTGGGTAACTCCATTGAACAAGAGTGAACGA
TGGGCTGGTGGGCTGTTTGTCTATGAGAGCCACGGCGAGGATACTCTTGCTGTTTGGTCC
AAGAGGAATAGAGCGATCCAAGGAAGAGATATAGTGCTGTGGTACACAGTGGGGTTCCAT
CACGTTCCATGCCAGGAGGATTTTCCAATAATGCCCACATTAAGCGGCAGCTTTGATCTG
AAGCCAAGTAATTTCTTTGAGAGAAATCCTATCCTAAAAACACTCCCAAATTACCCCTCC
CAGCTGCCCAAGTGTTCAATTGGCAGCCATGTTGACGAGAGGTAG

3) Polyketide synthases (PKS)

>HsPKS1 (ABI94386.1)
MTIKGSGSAAFEGTRLCPRVIKPDGPATILAIGTSNPTNIFEQSTYPDFFFDVTNCNDKTELKKKFQRICDKSGIKK
RHFHLTDEILRKNPSICKFKEASLDPRQDIAVLEVPKLAKEAAISAIKQWGQPKSKITHLVFATTSGVDMPGADFQ
LAKLLGLRPTVKRVMLYQQGCYAGATVLRVAKDLAENNKGARVLVACSEVTAVTFRAPSETHLDGLVGSALFGD
GAAALIVGSDPVPQEEKPLFEIHWAGEAVLPDSDGAINGHLREAGLIFHLLKDVPGLISKNIDKVLAEPLEYVHFP
SYNDMFWAVHPGGPAILDQIEAKLGLSTDKMQASRDVLASYGNMSSASVLFVLDQIRKNSEELHLPTTGEGFE
WGFVIGFGPGLTVETLLLRSINI

>HsPKS2
MPATELAASLKANGNGISSSFRTSREKPDGPASVLAIGVANPLHVFEQSTYPDFFFDITSCNEKTELKKKFQRICD
RSGIRKRHFFLTEEILKANPSMCNYMAASLDVRQDIAVAEAPKLAKEAALKAIKEWGQPKAKITHLVFATTSGVD
MPGADFKLTKLLGLRPDVKRVMLYQQGCYAGATVTRIGKDLAENNKGSRVLVVCSEVTAVTFRAPSETHLDGLV
GSALFGDGAAALILGSDPLPEVEKPVFEIHWAGELILPDSDGAINGHLKEAGLVFHLLKDVPGLISKNIEKILSDAL
KYAGSPEYNNIFWAVHPGGPAIILDQMEQNLKLTKDKLTSTRDILADYGNMSSASVLFVLNQLRKRSQELHLATT
GEGCEWGVMIGFGPGLTVETLVLRSTQL

>HsPKS3

MLHQQYATEASVADDFSSCQIKPDGQATVLAIGTANPPHVIEQSAFPDFPDFYFNVTNCSGKSELKKKFQRSGV
KRRHVFLTEEILKANPSMCTYMASSLNVRQEIANLEVPKLAKEAALKAIEEWGQPKSKITHLVFATSNGNAMPG
ADFKLVKLLGLRPDVKRVMLYQQGCFAGASVTRIGKDLAENNKGARVLAVCSEITAFTFQAPSDTHFPNLINSAL
FGDGAAALILGSHPIPGLEKPIFEIHWAGQTIVPDSDDAVAGRLTEAGMVFLLMKGLSQLISANIETILSEALRKA
GSPGYKDIFWAVHPGGLAIIDALERKLKLTADKMASAREILAAYGNMSSPSVLFVLDQLRKKSQNMKFSTTGEG
CEWGVMMGFGPGFTLEVLVLKSILLHELTSTDNRYLESS

>Unigene393

MTIKGSGSAAFEGTRLCPRVIKPDGPATILAIGTSNPTNIFEQSTYPDFFFDVTNCNDKTELKKKFQRICDKSGIKK
RHFHLTDEILRKNPSICKFKEASLDPRQDIAVLEVPKLAKEAAISAIKQWGQPKSKITHLVFATTSGVDMPGADFQ
LAKLLGLRPTVKRVMLYQQGCYAGATVLRVAKDLAENNKGARVLVACSEVTAVTFRAPSETHLDGLVGSALFGD
GAAALIVGSDPVPQEEKPLFEIHWAGEAVLPDSDGAINGHLREAGLIFHLLKDVPGLISKNIDKVLAEPLEYVHFP
SYNDMFWAVHPGGPAILDQIEAKLGLSTDKMQASRDVLASYGNMSSASVLFVLDQIRKNSEELHLPTTGEGFE
WGFVIGFGPGLTVETLLLRSINI

>Unigene394

MTIKGSGSAAFEGTRLCPRVIKPDGPATILAIGTSNPTNIFEQSTYPDFFFDVTNCNDKTELKKKFQRICDKSGIKK
RHFHLTDEILRKNPSICKFKEASLDPRQDIAVLEVPKLAKEAAISAIKQWGQPKSKITHLVFATTSGVDMPGADFQ
LAKLLGLRPTVKRVMLYQQGCYAGATVLRVAKDLAENNKGARVLVACSEVTAVTFRAPSETHLDGLVGSALFGD
GAAALIVGSDPVPQEEKPLFEIHWAGEAVLPDSDGAINGHLREAGLIFHLLKDVPGLISKNIDKVLAEPLEYVHFP
SYNDMFWAVHPGGPAILDQIEAKLGLSTDKMQASRDVLASYGNMSSASVLFVLDQIRKNSEELHLPTTGEGFE
WGFVIGFGPGLTVETLLLRSINI

>CL2724.2

MTIKGSGSAAFEGTRLCPRVIKPDGPATILAIGTSNPTNIFEQSTYPDFFFDVTNCNDKTELKKKFQRICDKSGIKK
RHFHLTDEILRKNPSICKFKEASLDPRQDIAVLEVPKLAKEAAISAIKQWGQPKSKITHLVFATTSGVDMPGADFQ
LAKLLGLRPTVKRVMLYQQGCYAGATVLRVAKDLAENNKGARVLVACSEVTAVTFRAPSETHLDGLVGSALFGD
GAAALIVGSDPVPQEEKPLFEIHWAGEAVLPDSDGAINGHLREAGLIFHLLKDVPGLISKNIDKVLAEPLEYVHFP
SYNDMFWAVHPGGPAILDQIEAKLGLSTDKMQASRDVLASYGNMSSASVLFVLDQIRKNSEELHLPTTGEGFE
WGFVIGFGPGLTVETLLLRSINI
