# Supplementary material for: Global transcriptome analysis of Huperzia serrata and identification of critical genes involved in the biosynthesis of huperzine A
Source: BMC Genomics. 2017 Mar 22;18:245. doi: 10.1186/s12864-017-3615-8 (PMC5361696; doi:10.1186/s12864-017-3615-8)
Supplement: Supplementary file 5 — LDC’s, CAO’s, and PKS’s multiple-tissues specific expression patterns analysis by heat map. (PDF 63 kb) [file 12864_2017_3615_MOESM5_ESM.pdf]

LDC's (**A**), CAO's (**B**), and PKS's (**C**) multiple-tissues specific expression patterns analysis by heat map.

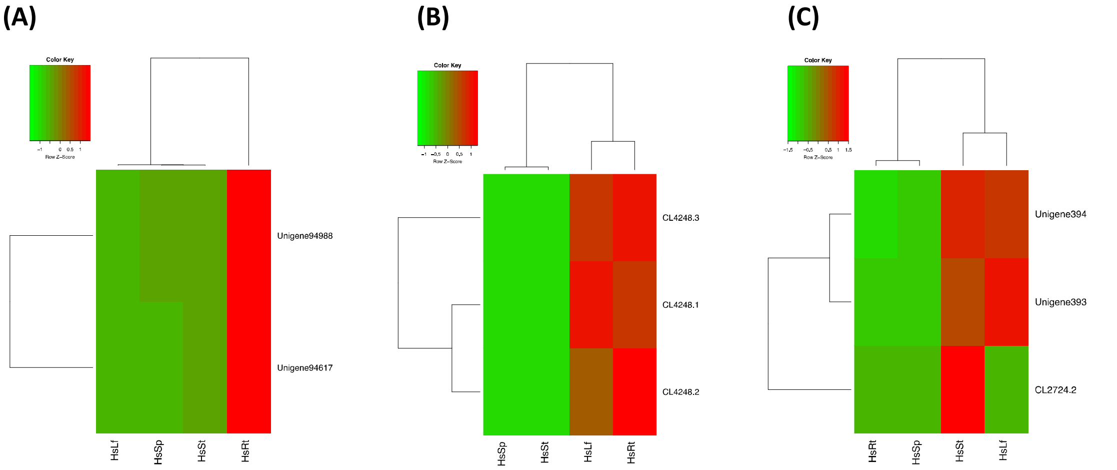

(D) Unigenes of LDC, CAO, and PKS identified in the four tissues.

Gene	Leaf	Stem	Root	Sporangia
**LDC**	**1) Unigene94988,<br>2) Unigene94617**	**1) Unigene94988,<br>2) Unigene94617**	**1) Unigene94988,<br>2) Unigene94617,<br>3) Unigene24553**	**1) Unigene94988,<br>2) Unigene94617,<br>3) Unigene24553**
**CAO**	**1) CL4248.1,<br>2) CL4248.2,<br>3) CL4248.3**	**1) CL4248.1,<br>2) CL4248.2,<br>3) CL4248.3**	**1) CL4248.1,<br>2) CL4248.2,<br>3) CL4248.3**	**1) CL4248.1,<br>2) CL4248.2,<br>3) CL4248.3**
**PKS**	**1) Unigene393,<br>2) Unigene394,<br>3) CL2724.1,<br>4) CL2724.2,<br>5) CL2724.3,<br>6) Unigene112370**	**1) Unigene393,<br>2) Unigene394,<br>3) CL2724.1,<br>4) CL2724.2,<br>5) CL2724.3,<br>6) Unigene112370,<br>7) CL8219.1**	**1) Unigene393,<br>2) Unigene394,<br>3) CL2724.1,<br>4) CL2724.2,<br>5) CL2724.3,<br>6) Unigene112370**	**1) Unigene393,<br>2) Unigene394,<br>3) CL2724.2**
