# Supplementary material for: Global transcriptome analysis of Huperzia serrata and identification of critical genes involved in the biosynthesis of huperzine A
Source: BMC Genomics. 2017 Mar 22;18:245. doi: 10.1186/s12864-017-3615-8 (PMC5361696; doi:10.1186/s12864-017-3615-8)
Supplement: Supplementary file 6 — Selected genes for LDC’s, CAO’s, and PKS’s phylogenetic analysis. (PDF 66 kb) [file 12864_2017_3615_MOESM6_ESM.pdf]

1) LDC data used for phylogenetic analysis was from the two references below.

a) Bunsupa S, Hanada K, Maruyama A, Aoyagi K, Komatsu K, Ueno H, Yamashita M, Sasaki R, Oikawa A, Saito K, Yamazaki M: Molecular Evolution and Functional Characterization of a Bifunctional Decarboxylase Involved in Lycopodium Alkaloid Biosynthesis. Plant Physiol. 2016; 171(4):2432-44.

b) Xu B, Lei L, Zhu X, Zhou Y, Xiao Y: Identification and characterization of L-lysine decarboxylase from Huperzia serrata and its role in the metabolic pathway of lycopodium alkaloid. Phytochemistry doi:10.1016/j.phytochem.2016.12.022. (*publish in process*).

2) Accession number of selected genes for CAO and PKS phylogenetic analysis

**No**	**CAO**	**PKS**
1	AAB30397.1	CAZ98522.1
2	KVH91911.1	CAQ52620.1
3	ACC83279.1	KXJ88015.1
4	AAC37012.1	AMW87979.1
5	CCA37360.1	KDP58758.1
6	YP_004995602.1	AGE44109.1
7	EGR49936.1	ADK13089.1
8	CCN29514.1	ADK45324.1
9	GAT26242.1	ADN26594.1
10	AKA31602.1	CBT77269.1
11	KMQ46717.1	O23674.1
12	WP_012544760.1	3ALE_A
13	AFJ46748.1	XP_013744791.1
14	GAM30363.1	
