# Supplementary material for: Global transcriptome analysis of Huperzia serrata and identification of critical genes involved in the biosynthesis of huperzine A
Source: BMC Genomics. 2017 Mar 22;18:245. doi: 10.1186/s12864-017-3615-8 (PMC5361696; doi:10.1186/s12864-017-3615-8)
Supplement: Supplementary file 8 — The amino acid sequences of 11 homologues identified as BBE. (PDF 33 kb) [file 12864_2017_3615_MOESM8_ESM.pdf]

**The amino acid sequences of BBE**

**>CL14177.1**
MAMAMEMEIAAGAKALGTTLLLLLLFLLWHRVRINPRLKLPPSPPGSWPLLGHLLHLRPLAHTALQAMSNIRG
PLITIRIGMLPCVVVSSAAAARECLITHGKVFASRPQPASWELFTDRWSTLTSAPYGDLWRSLRRIASVHLLNAK
MIASHRSVRVRYVGRMIEELRVEAAASPSGSVKALASIRRCIICIFAWVYFDLEVEDASSEAIVSEIDANIKEVNSAI
GTFSLGDCFPAIGWIFPTNRSYRQDLQRRTLALYRRLVDNVRQKMQDVRNSDSPTKSYAQILLSLQNSAEEPVL
SDDKILWLLQELMNAAVDTMANVLECGLAEITVNQQVQKKLQNEIMNTSTSGGEIQNVLQESDLQKLPYLEAV
VKETLRLHSSSPMGLPHATTEASQIAGFDIPAGTQILFLLRGLSTDPSFWEDPKKFRPERFVEQPMEANGQNM
NFIPFSAGKRICPGYSIAMLHLNYITGSLVQAFDWEAAPGGVDMSEKADSTVSLKTCFQPRIKPRII

**>CL14177.2**
MSNIRGPLITIRIGMLPCVVVSSAAAARECLITHGKVFASRPQPASWELFTDRWSTLTSAPYGDLWRSLRRIASV
HLLNAKMIASHRSVRVRYVGRMIEELRVEAAASPSGSVKALASIRRCIICIFAWVYFDLEVEDASSEAIVSEIDANI
KEVNSAIGTFSLGDCFPAIGWIFPTNRSYRQDLQRRTLALYRRLVDNVRQKMQDVRNSDSPTKSYAQILLSLQNS
AEEPVLSDDKILWLLQELMNAAVDTMANVLECGLAEITVNQQVQKKLQNEIMNTSTSGGEIQNVLQESDLQKL
PYLEAVVKETLRLHSSSPMGLPHATTEASQIAGFDIPAGTQILFLLRGLSTDPSFWEDPKKFRPERFVEQPMEAN
GQNMNFIPFSAGKRICPGYSIAMLHLNYITGSLVQAFDWEAAPGGVDMSEKADSTVSLKTCFQPRIKPRII

**>CL14177.6**
MAMAMEMEIAAGAKALGTTLLLLLLFLLWHRVRINPRLKLPPSPPGSWPLLGHLLHLRPLAHTALQAMSNIRG
PLITIRIGMLPCVVVSSAAAARECLITHGKVFASRPQPASWELFTDRWSTLTSAPYGDLWRSLRRIASVHLLNAK
MIASHRSVRVRYVGRMIEELRVEAAASPSGSVKALASIRRCIICIFAWVYFDLEVEDASSEAIVSEIDANIKEVNSAI
GTFSLGDCFPAIGWIFPTNRSYRQDLQRRTLALYRRLVDNVRQKMQDVRNSDSPTKSYAQILLSLQNSAEEPVL
SDDKILWLLQELMNAAVDTMANVLECGLAEITVNQQVQKKLQNEIMNTSTSGGEIQNVLQESDLQKLPYLEAV
VKETLRLHSSSPMGLPHATTEASQIAGFDIPAGTQILFLLRGLSTDPSFWEDPKKFRPERFVEQPMEANGQNM
NFIPFSAGKRICPGYSIAMLHLNYITGSLVQAFDWEAAPGGVDMSEKADSTVSLKTCFQPRIKPRII

**>Unigene678**
MALTTSSAWNQISWLLQSLPSLSIQPQAAAAALLLPIAAAAAAIAIYRSSSFCTSTRESCRLPPGPSGLPVVGSLF
WLSKLLRGSAKDLRAKYGPIVTVKVGSVPHIIIASAELAREVLIEKGSLFGSRPSNPALAVFSNNFRSINSSPSGPY
WRDLRTNLIVNFLSSTNVSAHTSTREDEVGNLISRIKLEAGENGEVSVLSNCRITILKILYRMCLDKSIDGDHLDEI
DKIYKEIFKSGKGSIADYVPWLWFIPKPYLKRQRQVRKRQTELLAPVFRACMKLRKEGNLKPSCYLSSLLALQSEE
GKPQFSEDDLITLISEVLNAGTDTTAQGLEWALIQLIIHPDKQAKLYEEISHIAEKSPVSEKDIERLPYLQAVVKETLR
MNPPGLFTLPHATLRPTKLSGYDIPTNARLLVHLYSIGNDPELWTMPDRFIPERFLDTNINITSGRYLKMIPFGAG
RRMCPGWGLAMLHMQLILARLVQSFKWSCANPDQLLSLIEIYKSSLIKTHNLKAIVSER

**>Unigene1166**
MREIFSQASAAVHGLILQLHHPSMAINLAIPGSNSTLNGVVYCLLVLLLLTALIQTKIFFFGSSKPSCLALPPGPRG
WPLVGCLLQLREITTSGVGDFVQKYGPIFTLQIGSKPIIVITDAETIHEAFVEKGSVFSSRPCDPSAALLTSNYRSIGS
APYGPRWRTLRKALVSGYFSSSKAFRDVREEAVQHLLQKLRDEAAHNAGIVVRVREIIKYGFFDLLLSLCFGKHAR
ESVVHDVAQILKHPRVLVEAGIDDFIPWLRFIPWRHTKQIQELRQEQLNVLLPLIREAQGLRQDGKLPGGSYIHS
LLAVDLEGKMLSDDELVTLCFEFLTGGTDTTATALEWAMANIISQPKIQAQIVSEMERVVGKRAAQEEDLDSLPY
LGAVAKETLRRHPPASFTLPHCLTQPCKLRGYNIPSNARVVASISSIANDPSIWENPTQFNPDRFLRSDHFDITGS

RKVTLIPFGTGRRICPGLGLATLNLNFILARLMQSFEWSTAAPDETVDLTPMFELTTVMKSPLRALIKAR

**>Unigene35401**
MQNMSEINHAEASNLDPFIARGLALTVMFGFLLLFLLKTIITKIFGLKPRLVQRLPPGPKGWPLVGCLFHFNTLFS
AGFQDWVKKYGPIITLPILSTPMIFIADADLVHEAFVEKGLCFASRPCIFSAALSSSNYRSINMALYGPIWRTLRKN
LVRFITSASKVSDFENLKEEVVQDLLRKFRDEAARNGGSVLQVRQICSYGFFDYALSLCFGKRPQERLVLEVAQL
MKKILLRSRSGIDDFVPWLRFVPWIRTKEHQELRQQQVNIMLPLIKEAERLRNDGKLSSSSYIDSLLSVDLQGQK
LSEDELLTLCSELLNAATDTISTTLEWAMANIINQPNIQAQIVSEIERVVGKRAVKEEDLGNLPYLEAVVMETLRR
HPPGYFTLPHVVTQPCKLQEYDIPLNANVFACIFSISNNPRVWGNPTEFKPDRFLNLDMDITGSKKITIIPFGTGR
RICPGLRLALLNLKFILARLIQSFEWSTTVPDETVDLTGTMEFVMVMKSPLRALIQNRC

**>Unigene35404**
MQNMSEINHAEASNLDPFIARGLALTVMFGFLLLFLLKTFITKIFGLKPRLVQRLPPGPKGWPLVGCLFHFNTLFS
AGFQDWVKKYGPIITLPILSTPMIFIADADLVHEAFVEKGLCFASRPCIFSAALSSSNYRSINMGLYGPVWRTLRK
NLVRFITSASKVSDFENLKEEVVQDLLRKFRDEAARNGGSVLQVRQICSYGFFDYALSLCFGRRPQERLLLEVAQL
MKKILLRSRSGIDDFVPWLRFVPWIRTKEHQELRQQQVNIMLPLIKEAERLRNDGKLSSSSYIDSLLSVDLQGQK
LSEDELLTLCSELLNAATDTISTTLEWAMANIINQPNIQAQIVSEIERVVGKRAVKEEDLGNLPYLEAVVMETLRR
HPPGYFTLPHVVTQPCKLQEYDIPLNANVFACIFSISNNPRVWGNPTEFKPDRFLNLDMDITGSKKITIIPFGTGR
RICPGLRLALLSLKFILARLIQSFEWSTTVPDETVDLTGTMEFVMVMKSPLRALIQNRC

**>Unigene35405**
MQNMSEINHAEASNLDPFIARGLALTVMFGFLLLFLLKTIITKIFGLKPRLVQRLPPGPKGWPLVGCLFHFNTLFS
AGFQDWVKKYGPIITLPILSTPMIFIADADLVHEAFVEKGLCFASRPCIFSAALSSSNYRSINMGLYGPVWRTLRK
NLVRFITSASKVSDFENLKEEVVQDLLRKFRDEAARNGGSVQVRQICSYGFFDYALSLCFGRRPQERLLLEVAQL
MKKILLRSRSGIDDFVPWLRFVPWIRTKEHQELRQQQVNIMLPLIKEAERLRNDGKLSSGSYIDSLLSVDLQG
EKLSEDELLTLCSELLNSATDTISTTLEWAMANIINQPKIQAQIVSEIERVVGKRAVKEEDLDNLPYLEAVVKETLRR
HPPGYFTLPHVVTQPCKLQEYDIPLNANVFACIFSISNNPRVWGNPTEFKPDRFLNLDMDITGSKKITIIPFGTGR
RICPGLRLALLNLKFILARLIQSFEWSTTVPDETVDLTGTMEFVMVMKSPLRALIQNRC

**>Unigene25120**
MAEINHAEAYSLIPYITTTRINELNIVLCLLVLLLFTIYVRKIFRFKHSFQLKLPPGPKGWPFVGCLFEFRSYFTSGFE
DLVQKYGPVFTLQLVSTPMIFITDADIIHEAFVEKGSIFASRPCGGPSAALFSSNYRNISSALYGPVWRTLRKNFVS
GVMNSSLKALNFRNVREEVVQGLLRRFRDEAAQNCGIVRVRQIIRYGIFELLLSLCFGKHAEERVVLDVAQVLKQ
ILLQYSSGSINDFIPWLRFIRWKQTKERIQLRQKQVKIILPLVKEAQRLHRDGKLTGGSYIDSLLSVDLQGKKLSDDE
LVTLCSELLVGGMDSTVTSLEWAIANIVSHPHAQSQIVSEIERVVGKKNVKEEEIENLRYLDAVVKETLRRHPPGY
VTLPHAVSQPCKLRGYDIPLNARVFASISSISTDPRIWDNPTNFKPERFLSSDFDITGSKNITMIPFGTGRRI

**>Unigene25121**
MQVMDEINHTKAHSLNPSITRSNSLNAVYCLLVLLLLTAFIRKIFGFKPSYQKLPPGPKGWPLVGCLFQFRRYFISG
FGDLVQEYGPIFTLQLVSTPMIFITDADIIHEAFVEKGSIFASRPCGGPSAALFSSNYRNISSALYGPVWRTLRKNFV
TGIMSSFRASNFRNVREEVVQDLLRRLSDEAAQNCGIVRVRQIIRYGIFELLLSLCFGKRAEERVVLDVAQVLKQIL
LRSRNGIDEFIPWLRFIPSKRTKERQQLRQQQVNVLLPLIKEAQRLRQDGNLLGGSYIDSLLAVDLQGKKLSDDEL
VSLCSEFLNAGSDTTTTALEWAIANIISRRHVQAQIKSEIERAVGNRAAKEEDIDNLPYLEAVVKETLRRHPPGYFT
LPHAVSQPCKLQGYDIPLNASVLACVSSISNNPRIWDDPTKFKPERFLSSDFDITGSKKITMIPFGTGRRICPGMG

LAMLHLNFILARLIQSFEWSTTAPDETIDLTPTFEFTVVMKSPLKALIINR

**>Unigene30042**

MAEIVNVAESYGLITTTPSINGLNIVLCLFVLLLFTIFIRRIFGFKLSSQKLPPGPKGWPVVGCLFQLRRYLTLELEDL
VQKYGPIITLQIVSTPIILIADADIVHEAFAEKGSNFVSRPHVASAALLSSNFKTVSMSPYGPVWRTLRKHLTTAIM
RSSKASSDLKNVREEVAQELLLGRFRDEAAQNGGIVQVRQVFKYGVFELLLSICFGKHAEERVVLDVAQILEQILI
RSSSLGIDAFLPWLSFFAWNRTKKHDQQLRQKQLNLILPLVKEAQMLRQDGKLAGGSYIDSLLEVDLQGEKLSD
DELVTLCSEFLIAATDSTVTTLEWAMANIISHPHIQSEIVNEIERVVGKRAAKEEDIENLPYLKAVVKETLRRHPPG
YFTFPYTVSQPCKLQGYDIPLNALVFASISSISTDPRIWDNPTEFKPERFLSSDFDISGSKKITMIPFGTGRRICPGM
GLATLHINFILARLVQSFEWRTADPGEPIDFTPILEVTTQMKSPLRALISNR
