# Supplementary material for: Global transcriptome analysis of Huperzia serrata and identification of critical genes involved in the biosynthesis of huperzine A
Source: BMC Genomics. 2017 Mar 22;18:245. doi: 10.1186/s12864-017-3615-8 (PMC5361696; doi:10.1186/s12864-017-3615-8)
Supplement: Supplementary file 9 — The amino acid sequences of 12 homologues identified as SLS. (PDF 35 kb) [file 12864_2017_3615_MOESM9_ESM.pdf]

The amino acid sequences of SLS

>CL9415.2
MDAIHEVMWAAFVAWIVGVLIHMWWKPLRLRKHYEKQGIRGPPFRPVLGNLPELRGLTESVPPQASSLTELS
MRRVAPELVSFFDSYGKVSIHEVGRKTRLLLVDVKLAREVLSTKAAFYQKADLSRESFWRLLGKGLVFSEGELWH
QQRRILSTGFRHQYLKDFCNLMKDACDNILVRWKKNWEESNNCAGVEIEVSKEMSMLTLDIMSRALFGSNEG
GKKHASDGALAFECLTGLFISSMRSSYSYKRLLPGYSIFPTIENWSSRRREKFVNSLLENIIRSRMATKEAAHDRVK
NEQGDDLLGVMLDALYEGSPTMSVPQLIHEAKTLFFAGYATTSLICTWCLTVLSMHQEWQEKARKEVQNVFG
DDITPDIKLLGKLKIVGMILNETLRLYPPLPILSRQCTKAHHIGDISMLPNTIVVIPTVLFHQSEELWGDDVSVFKPE
RFENKAAKDFPGFMPFGGGPRSCLGINFATIEVKIIISMLLRNFSFSLSPGYCHAPGLVSSMRPQFGVPLILTRL

>CL9415.5
MDANQAMMWAAFVAWIVGMFYHELWWKPLRLRKHYEMQGIRGPPFRPVLGNLPELRRLTDSVPPQASCF
TELSMHRVAPELVSFFSSYGKVSLHEVGRKMRLLLVDENLAREVLVNKAALYQKADLNREVFWRLLGKGLVFLEG
DFWHQQRRILLTGFRNQYLKGFSKFMNDACENLVVRWKKNWEESKNCASVEIEVSKEMSLLTLDIISRALFGSS
EGGKKHASDGAIAFERLSVLFFSCMRGSYSYKRLLPGYSLFPTIENWSSRGHEKFVNSLLEGIIRSRMVKKKARVV
KVKNEQEDDLLGVMLDALYEGSPTMSVPQLIHEAKTLFFAGHATTAVVLTWCLTLLSMHQEWQEKARKEVQN
VFGDDTIPDIKLLGKLKIVGMILNETLRLYPPLPILSRQCMKAHHIGNISMLPGTLVVIPVVMFHQSRELWGDDV
SEFKPERFQNKEAIDFSSFMPFGGGPRSCLGINFATIEVKIIISMLLRNFSFSLSPGYCHAPGLVSSMRPQFGVPLIL
TRL

>CL9415.8
MDAIHEVMWAAFVAWIVGVLIHMWWKPLRLRKHYEKQGIRGPPFRPVLGNLPELRGLTESVPPQASSLTELS
MRRVAPELVSFFDSYGKVSIHEVGRKTRLLLVDVKLAREVLSTKAAFYQKADLSRESFWRLLGKGLVFSEGELWH
QQRRILSTGFRHQYLKDFCNLMKDACDNILVRWKKNWEESNNCAGVEIEVSKEMSMLTLDIMSRALFGSNEG
GKKHASDGALAFECLTGLFISSMRSSYSYKRLLPGYSIFPTIENWSSRRREKFVNSLLENIIRSRMATKEAAHDRVK
NEKEDDILGVMLDALYEGSPTMSVPQLIHESKTLFFAGHATTSVILTWCLTLLSMHQEWQEKARNEMQNVFGD
DITPDIKLIGKLKIMGMILNETLRLYPPVSTLSRKCMKAHHIGSISMLPDTLVVIPTVLFHQSEELWGDDVSVFKPE
RFENKEAKDFLGFMPFGGGPRTCLGINFATIEAKFVLSMLLRNFNFSLSPSYRHAPGLASSMRPQFGVPLIVTRL

>CL9415.12
MLTYGSFMRGCIGAGKVSIHEVGRKTRLLLVDVKLAREVLSTKAAFYQKADLSRESFWRLLGKGLVFSEGELWH
QQRRILSTGFRHQYLKDFCNLMKDACDNILVRWKKNWEESNNCAGVEIEVSKEMSMLTLDIMSRALFGSNEG
GKKHASDGALAFECLTGLFISSMRSSYSYKRLLPGYSIFPTIENWSSRRREKFVNSLLENIIRSRMATKEAAHDRVK
NEQGDDLLGVMLDALYEGSPTMSVPQLIHEAKTLFFAGYATTSLICTWCLTVLSMHQEWQEKARKEVQNVFG
DDITPDIKLLGKLKIVGMILNETLRLYPPLPILSRQCTKAHHIGDISMLPNTIVVIPTVLFHQSEELWGDDVSVFKPE
RFENKAAKDFPGFMPFGGGPRTCIGSNFAVIEVKTILSMLLKNFNFSLSPGYCHSPGLASSMRPQFGVPLIVTRL

>CL10516.1
MTGFQCFVLALLIALCAVMLLICNRIVVFVSIYWWKPVIMRRLLMKQGLEGPPPKFMVGNVHEVAEMKKKIAA
TDMKIGDHDIMSRVCPYYCEWAEKYGKRLVFWWGIEPRITVTEPEIIKEILATKAGHFGKSLLQQKGGVALLGN
GLIMANGEDWAHRRRIVSRAFQLEKLKGMVASMVQSTNQITDKWDTLIRNNEGKIAEIDVYEQFAIVTADIIAR
TSFGSSFEQGKQVFHLLRSLQKVFAESNRFVWLPGSRFLPTPTNRKLSSLKKAMAKSLESIVDARRRSASLDADP
GSFGNDLLGLMLAESAGTVPSKSSQKRFKFSTEELVEECKTFFFVGHETTLILLTWTMMLLALNPEWQEKARME

VQENFKGRLEPDADMLSKLKIVTMILNESLRLYPPAPVLVREAFKDMQLGDMFVPKGCTFWMPILAIHHDPQL
WGPDPMEFRPERFAEGISKACKRPNEFMPFSFGPRACVGQTFAMMEAKTVLAMMLLRYRFSLSSSYRHAPVT
SITIKPKYGMPLLVEHI

>CL10516.2
MTGRQCLALTVLLVVCAIILVLWNKFLIFVSNYWWKPFVMRRLLMKQGVDGPPPSFMVGNMHEMTEMRNK
VAETDMEVGNHNIMSRVCPYYCEWAKKYGKRLVFWWGIEPRITVSEPEIIKQILATKAGHFGKSALQQKGGAA
LLGNGLIMANGEDWAHRRRIVSRAFQLEKLKGMVPSMVQSTNEVLEKWDAVIRMNGGENAEIDVYEQFALV
TADVIARTSFGSSFEQGKQVFQLLRSLQKVFVESNRFVWLPGSRFLPTATNRKVSALKKAMARSLEDIVDARRRS
VGNNADAASYGNDLLGLMLAASAGTPSKDTTKSFKFSTEELIEECKTFYFVGHETTLILLTWTMMLLALNPEWQ
EKARTEVQESFKGRSELDVDMLSKLKTVTMVLNESLRLYPPAPVLVREAFKDMTLGDMFVPKGCTFWMPILAI
HHDSQLWGPDSMEFKPERFAEGISKACKRPNDFMPFSFGPRACVGQTFAMMEAKTVLAMVLLRYKWCLSSS
YRHAPVTSITIKPKHGMPLIVERI

>CL11443.2
MYHAHAHYNMGMSTRSDVLLPMLLAASTAILMFACNATIKLLRYYCLQPWNLARALQAQGLKGPAPRFLLGNI
SQIVEMRNEKEIHDMKTGDHNILSRICPYYCQWSEEYGKRFVFWWGTEPRIPATDSESLKQILCTKSEYFGKSSI
QLKGGILLLGNGLIFANGADWAKRRQIVGPAFHVDKLKQMVPLMVNCVSSMLQKWDQILRTTEGSVEIDVYD
QFAGLTSDVIVRTAFGSGFRDGKKVFQILKSLEKIFSDYNRYIWVPGCRLLPTPLNRKASRLQQEMQSSLQAIIEA
RRASDTCERGYSPKRPSDLLGLLMAESNKNHHGPEQKFGSNEMLQECRTFFFVGHETTFLLLTWTVMLLALHP
YWQEEARAEVVQVCKGSVPHADLLNRLKIVGMILNESLRLYPPAPTLLRTALDDVEIGSMHVPKGTTFWIPILAI
HHNPALWGADANEFKPERFSDGNTHSYKHSLSFLPFSFGSRSCVGQTFALMEAKVVLVMLLQNYRFLLDIQAP
ALAPAWIFNELHKPSCPSKSTPRA

>CL160.1
MFVEWVLSVAGVAVAWILILWVAKIVRDLWWRPLQIYKRLHAQGVQGPPFRAVVGNQPEFVRMLAAAASSPI
KLLSHEIVPHVLPYYKTWSKIYGDMFTYAYGSEVRLTITDPELMKEILSNRFGHFPKVASTPAARDLLGDNGLVLAT
GEKWAQERRILSHGFHVDKLKAMVETMAELTTKMLNDWERRIVAAKQPKDVEIEVHEQYQNLTADIISHTAFG
SSYEAGKKVFQLQYLQARMVRELAQSIRFPGRSLFRIAKYLRGRKIRKIIEENLLDIIRKRLQQTPENDIQHDDLLG
VMVCAYKGQLRGNQKNLRMSIQDIVDECKTFYFAGHETTTSLLTWTTMLLALHPSWQERVRAEVMSVCGSN
NPTGDMLTQLKLMGNVLNESLRLYSPATVLVRCTDRDMKLGKHEQLLIPKGTVLSLLVIAMLHDKKVWGEDAD
TFNPERFSEGIANASTHPSAFMPFSMGPRNCIGQVFALMEAKVVLCKLLQRFSFELSAAYVHSPSQFGLMRPNF
GMQIHATPIQC

>CL160.2
MFVEWVLSVAGVAVACILILWVAKIVRDLWWRPLQIYKRLHAQGVQGPPFRAVVGNQPEFVRMLAAAASSPI
KPLSHEIVSHVLPHYKTWSKIYGEMFTYAYGSEVRLTITDPELMKEILSNRFGHFPKVTSNPAARDLLGDNGLVLA
TGEKWAQERRILSHGFHVDKLKAMVETMAELTTKMLNDWERRIVAAKQPKDVEIEVHEQYQNLTADIISHTAF
GSNYEAGKKVFQLQYLQARMVRELAQSIRFPGRSLFRIAKYLRRRKIRKTIEENLLDIIGKRLQQTPENDIQHDDL
LGVMVCAYKGQLRGNQKNLRMSIQDIVDECKTIYFAGHQTTTSLLAWTTMLLALHPSWQERVRAEVMSVCG
SNNPNGDMLTQLKLMGNVLNESLRLYSPLPVMVRCTDRDMKLGKHEQLLIPKGTVLSLLVIAMLHDKKVWGE
DADTFNPERFSEGIANASTNPSAFMPFSMGPRNCIGQVFALMEAKVVLCKLLQRFSFELSAAYVHSPSQLGLM
RPNFGMQIHATPMPFTTS

>CL160.3
MFVEWVLAVAGVAVACILILWVAKIVRDLWWRPLQIYKRLHAQGVQGPPFRAVVGNQPEFVRMLAAAASSRI
KLLSHEIVPHVLPHYKTWSKIYGDMFTYAYGSEVRLTITDPELMKEILSNRFGHFPKVTSNPAARDLLGDNGLVLA
TGEKWAQERRILSHGFHVDKLKAMVETMAELTTKMLNDWERRIVAAKQPKDVEIEVHEQYQNLTADIISHTAF
GSNYEAGKKVFQLQYLQARMVRELSQSIRFPGRSLFRIAKYLRRRKIRKTIEENLQDIIRKRLQQTPENDIQHDDL
LGVMVCAYKGQLRGNQKNLRMSIQDIVDECKTFYFAGHETTTSLLTWTTMLLAFHPSWQERVRAEVMSVCGS
NNPTGDMLTQLKLMGNVLNESLRLYSPATVLVRCTDRDMKLGKHEQLLIPKGTVLSLLVIAMLHDKKVWGEDA
DTFNPERFSEGIANASTHPSAFMPFSMGPRNCIGQVFALMEAKVVLCKLLQRFSFELSAAYVHSPSQLGLMRP
NFGMQIHATPMPFTAS

>CL160.7
MFVEWVLAVAGVAVACILILWVAKIVRDLWWRPLQIYSRLHAQGVQGPPFRAVVGNQPEFVRMLAAAASSTI
KPLSHEIVSHVLPHYKTWSKIYGDMFTYAYGSEVRLTITDPELMKEILSNRFGHFPKVTSNPAARDLLGDNGLVLA
TGEKWAQERRILSHGFHVDKLKAMVETMAELTTKMLNDWERRIVAAKQPKDVEIEVHEQYQNLTADIISHTAF
GSNYEAGKKVFQLQYLQARMVLELAQSIRFPGRSLFRIAKYLRRRKIRKTIEENLLDIIGKRLQQTPENDIQHDDLL
GVMVCAYKGQLRGNQKNLRMSIQDIVDECKTFYFAGHETTTSLLTWTTMLLAFHPSWQERVRAEVMSVCGS
NNPTGDMLTQLKLMGNVLNESLRLYSPATVLVRCTDRDMKLGKHEQLLIPKGTVLSLLVIAMLHDKKVWGEDA
DTFNPERFSEGIANASRHPSAFMPFSMGPRNCIGQVFALMEAKVVLCKLLQRFSFELSAAYVHSPSQLGLMRP
NFGMQIHATPMPFTAS

>CL160.9
MFVEWVLAVAGSVVATCILFLWVVKIVRDLWWRPLQIYKSLHAQGVQGPPFRAVVGNQPEFDRMLAAAASS
RIKLPSHEIVPHVLPHYKTWSTIYGDMFTYAYGSEVRLTITDPELMKDILSNRFGHFPKVPPNPASRDLLGENGLV
LATGEKWAQERRILSHGFHVDKLKAMVETMAELTTKMLNVWERRIVAAKQPKDVEIEVHEQYQNLTADIISHT
AFGSSYEAGKKVFQLQYLQAEMVRELSHSIRFPGRSLFRIAKYLRRRKIRKTIEENLQDIVRKRLQQTPENDIQHD
DLLGVMVCAYKGQLRGNQKNLRMSIQDIVDECKTFYFAGHETTSSLLTWATMLLALHPSWQERVRAEVTSVC
GSNNPTGDMLTQLKLMGNVLNETLRLYSPAIVLGRCTDRDMKLGKQQLVIPKGTELSLLLIAMLHDKKVWGED
ADNFNPDRFAEGVANASRHPSAFMPFSMGPRNCIGQVFAQMEAKVVLCKLLQRFSFELSATYVHSPTQFGLLR
PSFGMQILATPIHCYR
