# Supplementary material for: Global transcriptome analysis of Huperzia serrata and identification of critical genes involved in the biosynthesis of huperzine A
Source: BMC Genomics. 2017 Mar 22;18:245. doi: 10.1186/s12864-017-3615-8 (PMC5361696; doi:10.1186/s12864-017-3615-8)
Supplement: Supplementary file 10 — qRT-PCR expression analyses of HsLDC, HsCAO, HsPKS, and CYP450s. (PDF 36 kb) [file 12864_2017_3615_MOESM10_ESM.pdf]

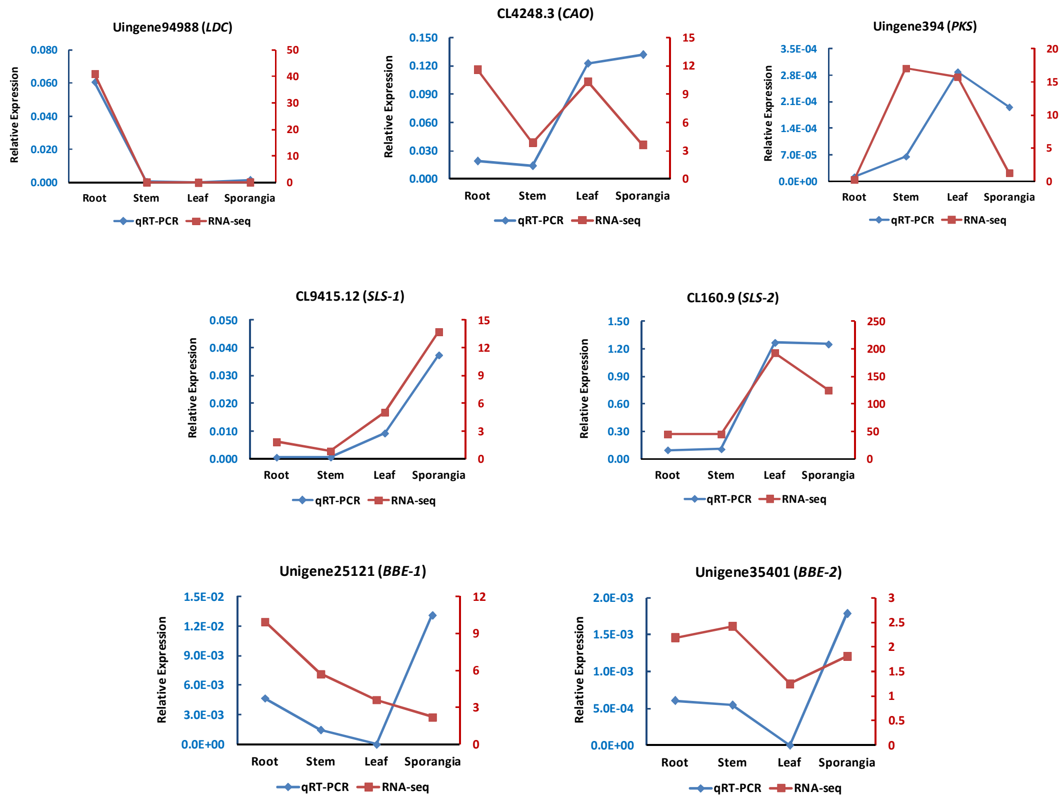

Real-Time PCR expression analyses of *HsLDC*, *HsCAO*, *HsPKS*, and *CYP450s* (SLS and BBE classes)
qRT-PCR was used to validate several of the differentially expressed genes identified by RNA-Seq in the four tissues of *H. serrata*. Using the internal control, tubulin (Unigene50132), seven selected genes involved in HupA biosynthesis, including HsLDC, HsCAO, HsPKS and CYP450s (SLS and BBE classes), were evaluated.

Primers for qRT-PCR

Gene name	Gene ID	Forward primer (5' - 3')	Reverse primer (5' - 3')
**LDC**	Unigene94988	CACGGATGGAGATCATGACAAGT	CGCCGAGGTCTAGAACGAAG
**CAO**	CL4248.3	CTCTGGGTTTTGTATCCTTCTGC	TGCTTGTCTTTTGGCACCCT
**PKS**	Unigene394	CATTACTTATAATAAGAAAAGTATGGGACA	GCAGTATTTCACGTATATGTTACAATAACT
**CYP450 (SLS-1)**	CL160.9	CCTGTGGGTGGTGAAGATTGTAA	GTTTTATAGTGAGGAAGCACGTGAG
**CYP450 (SLS-2)**	CL9415.12	TATGGATCATTCATGCGTGGC	AGATTCTTCGCTGCTGATGCC
**CYP450 (BBE-1)**	Unigene25121	CACACGAAAGCACATAGTTTAAATCC	CGAATCCCGATATGAAATACCTCC
**CYP450 (BBE-2)**	Unigene35401	ATTTAGATCCGTTCATCGCTCG	GAAGTGGAATAAGCATCCGACG
**Tubulin**	Unigene50132	AGTCTAGCGTCTGCGATATTG	CCATCTCATCCATACCTTCTCC
